# Full efficacy and long-term immunogenicity induced by the SARS-CoV-2 vaccine candidate MVA-CoV2-S in mice

**DOI:** 10.1038/s41541-022-00440-w

**Published:** 2022-02-09

**Authors:** Adrián Lázaro-Frías, Patricia Pérez, Carmen Zamora, Pedro J. Sánchez-Cordón, María Guzmán, Joanna Luczkowiak, Rafael Delgado, José M. Casasnovas, Mariano Esteban, Juan García-Arriaza

**Affiliations:** 1 Department of Molecular and Cellular Biology, Centro Nacional de Biotecnología (CNB), Consejo Superior de Investigaciones Científicas (CSIC), 28049 Madrid, Spain; 2 Centro de Investigación Biomédica en Red de Enfermedades Infecciosas (CIBERINFEC), Madrid, Spain; 3 Pathology Department, Centro de Investigación en Sanidad Animal (CISA), Instituto Nacional de Investigación y Tecnología Agraria y Alimentaria (INIA), Consejo Superior de Investigaciones Científicas (CSIC), 28130 Valdeolmos, Madrid Spain; 4 Department of Macromolecular Structures, Centro Nacional de Biotecnología (CNB), Consejo Superior de Investigaciones Científicas (CSIC), 28049 Madrid, Spain; 5 Instituto de Investigación Hospital Universitario 12 de Octubre (imas12), 28041 Madrid, Spain; 6 Universidad Complutense School of Medicine, 28040 Madrid, Spain

**Keywords:** Live attenuated vaccines, Viral infection

## Abstract

Two doses of the MVA-CoV2-S vaccine candidate expressing the SARS-CoV-2 spike (S) protein protected K18-hACE2 transgenic mice from a lethal dose of SARS-CoV-2. This vaccination regimen prevented virus replication in the lungs, reduced lung pathology, and diminished levels of pro-inflammatory cytokines. High titers of IgG antibodies against S and receptor-binding domain (RBD) proteins and of neutralizing antibodies were induced against parental virus and variants of concern, markers that correlated with protection. Similar SARS-CoV-2-specific antibody responses were observed at prechallenge and postchallenge in the two-dose regimen, while the single-dose treatment does not avoid vaccine breakthrough infection. All vaccinated animals survived infection and were also protected to SARS-CoV-2 reinfection. Furthermore, two MVA-CoV2-S doses induced long-term memory S-specific humoral and cellular immune responses in C57BL/6 mice, 6 months after immunization. The efficacy and immunological benefits of the MVA-CoV2-S vaccine candidate against COVID-19 supports its consideration for human clinical trials.

## Introduction

The COVID-19 pandemic, responsible for one of the major tragedies caused by an infectious agent in the course of one year (about 238 million infections and 4.8 million deaths by October 2021), outweighs the human deaths by other infectious diseases. In an unprecedented record time, numerous vaccine candidates against COVID-19 have been developed (mainly based on the SARS-CoV-2 spike (S) protein) and evaluated in phase I/II/III clinical trials, leading to the approval of some of them by the regulatory agencies. The most advanced vaccines in mass vaccination are based on adenovirus vectors (AstraZeneca, Janssen) and on mRNA (Pfizer/BioNTech and Moderna). It is remarkable the high efficacy of these vaccines, which ranges from 62-95% protection against virus infection and/or clinical disease^1–3^.

Development of novel vaccine candidates against COVID-19 with different modes of action is of great interest. We and others have described the advantage of the poxvirus vector modified vaccinia virus Ankara (MVA) as an immunizing agent against SARS-CoV-2, probing high immunogenicity and efficacy in mouse and macaque models^4–8^. Moreover, MVA-based vaccines against prevalent and emerging human viral diseases are highly immunogenic and effective in animal models^9–12^. Due to the complexity of the poxvirus vectors in comparison with other non-replicating live attenuated vectors, it is essential to provide accurate knowledge on the immune parameters triggered by the MVA-based vector vaccines and its correlation with protection. Previously, we have reported that an MVA vector expressing a human codon optimized full-length SARS-CoV-2 S protein (termed MVA-CoV2-S or MVA-S in the abbreviated form) elicited potent adaptive S-specific CD4^+^ and CD8^+^ T-cellular and humoral immune responses in wild-type C57BL/6 mice immunized with either heterologous DNA/MVA or homologous MVA/MVA immunization regimens^5^. Moreover, one or two doses of MVA-CoV2-S controlled morbidity (weight loss) and mortality caused by SARS-CoV-2 infection in K18-hACE2 transgenic mice, being the two-dose regimen the most effective^5^.

Here, we provide an evaluation of the efficacy and immunogenicity of one or two doses of MVA-CoV2-S in K18-hACE2 transgenic mice challenged with SARS-CoV-2, as well as the demonstration of long-term memory S-specific T-cell and humoral responses in immunized wild-type C57BL/6 mice. Overall, our findings add further support to the benefits of the recombinant MVA-CoV2-S vector as a vaccine candidate against SARS-CoV-2, worth to move forward into clinical trials.

## Results

### Two doses of MVA-CoV2-S vaccine candidate prevented SARS-CoV-2 virus replication, reduced lung pathology and levels of pro-inflammatory cytokines in infected K18-hACE2 transgenic mice

**Fig. 1:**
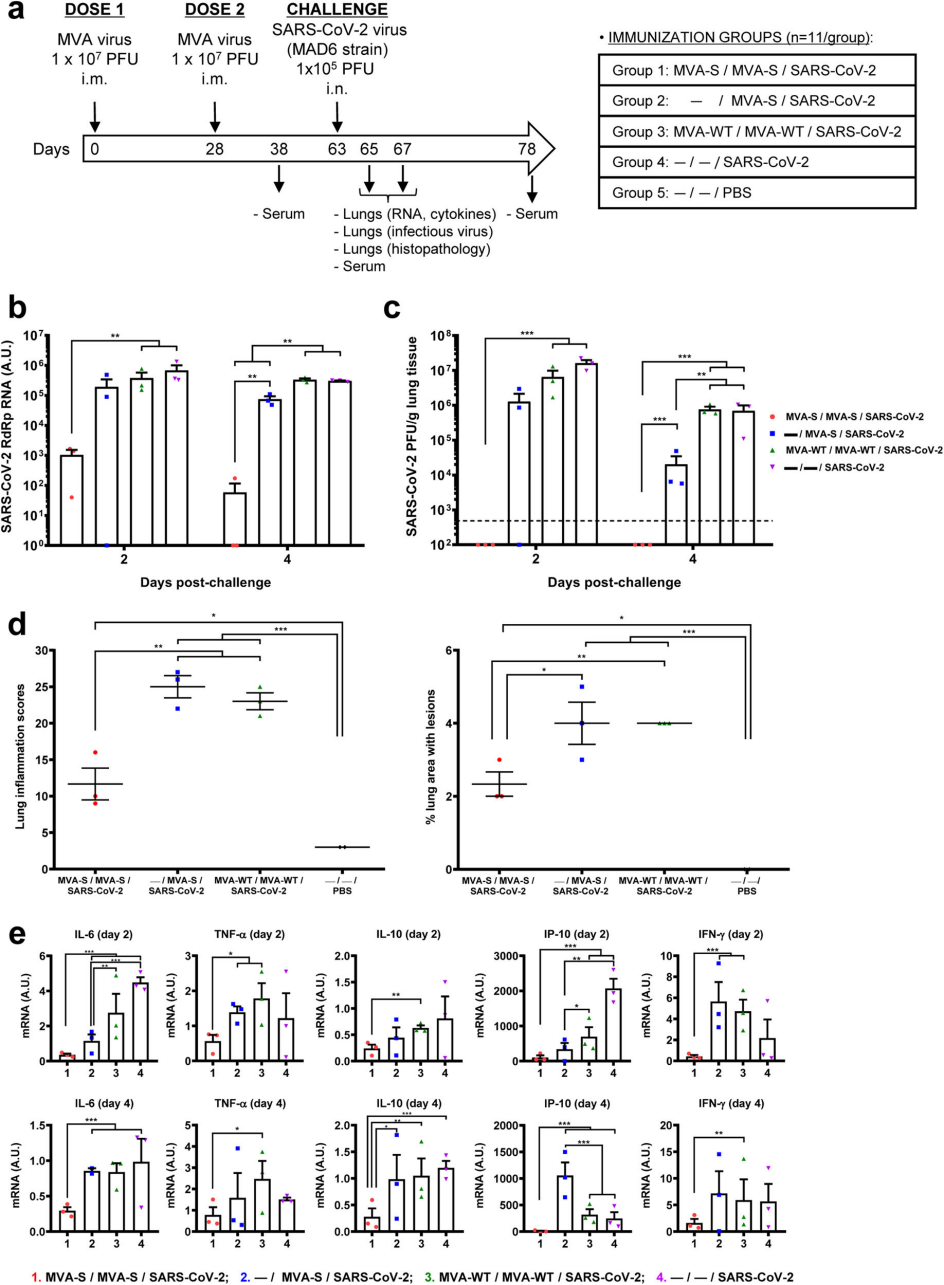
MVA-CoV2-S vaccine candidate prevented SARS-CoV-2 virus replication, reduced lung pathology and diminished levels of pro-inflammatory cytokines in K18-hACE2 transgenic mice. **a** Efficacy schedule. Female K18-hACE2 transgenic mice (*n* = 11 per group) were immunized by the intramuscular (i.m.) route with one or two doses of 1 × 10^7^ PFU of MVA-CoV2-S (also termed MVA-S). At 10 days after the last immunization (day 38) mice were bleed and serum samples were collected. Mice were challenged intranasally (i.n.) with 1 × 10^5^ PFU of SARS-CoV-2 (MAD6 isolate) at week 9 (day 63). MVA-WT-inoculated (group 3) and unvaccinated (group 4) mice were also i.n. challenged with SARS-CoV-2; unvaccinated and unchallenged mice were used as a negative control (group 5). At days 2 and 4 postchallenge 3 mice per group were sacrificed and lungs and serum samples collected as indicated. Serum was collected in mice alive at 15 days postchallenge (groups 1, 2, and 5). **b** Virus replication in lung samples. Genomic (RdRp) SARS-CoV-2 RNA detected by RT-qPCR at 2 (*n* = 3) and 4 (*n* = 3) days after virus infection. Mean RNA levels (in arbitrary units [A.U.]) and SEM from duplicates of each lung sample; relative values are referred to uninfected mice. **c** SARS-CoV-2 infectious virus in lung samples. Mean (PFU/g of lung tissue) and SEM from triplicates of each lung sample. **d** Lung inflammation scores and percentage of lung area with lesions. Examined in lung samples (*n* = 3) at 4 days postchallenge. Mean and SEM of cumulative histopathological lesion scores (left) and percentage of lung area affected by inflammatory lesions (right). **e** Identification of pro-inflammatory cytokines. mRNA levels detected by RT-qPCR in lungs obtained at 2 (*n* = 3) and 4 (*n* = 3) days postchallenge. Mean RNA levels (in A.U.) and SEM from duplicates of each lung sample; relative values are referred to uninfected mice. Student’s *t*-test: *P < 0.05; **P < 0.005; ***P < 0.001.

Here, we extended a previous efficacy study with MVA-CoV2-S vaccine candidate (also termed MVA-S) in K18-hACE2 transgenic mice^5^, susceptible to SARS-CoV-2 infection^13–16^. We further evaluated in detail the efficacy triggered after vaccination with one or two doses of MVA-CoV2-S, through analysis after SARS-CoV-2 infection of viral load, histopathology, and pro-inflammatory cytokine expression levels in lung samples. Thus, K18-hACE2 mice (*n* = 11/group) were intramuscularly immunized at weeks 0 and 4 with two doses of MVA-CoV2-S or at week 4 with one dose of MVA-CoV2-S, and then challenged 5 weeks later with a lethal dose of SARS-CoV-2 (MAD6 isolate, 10^5^ plaque-forming units (PFU)/mouse), by the intranasal route (Fig. 1a). Challenged mice primed and boosted with MVA-WT or unvaccinated, and unchallenged mice were used as control groups. Changes in body weight and mortality after SARS-CoV-2 infection were previously reported and showed that all mice vaccinated with two doses of MVA-CoV2-S did not lose body weight and survived, whereas mice immunized with one dose of MVA-CoV2-S lost body weight during the first 4 days postchallenge, but they recovered and survived^5^. In contrast, all mice inoculated with MVA-WT or unvaccinated and infected lost body weight and died at 6 days postchallenge^5^.

To examine the effect of MVA-CoV2-S vaccination on SARS-CoV-2 virus replication, three mice per group were sacrificed at days 2 and 4 after SARS-CoV-2 virus challenge, lungs were collected and processed, and presence of SARS-CoV-2 genomic RNA (Fig. 1b) and live infectious virus (Fig. 1c) was analyzed. Two doses of MVA-CoV2-S were highly effective to prevent SARS-CoV-2 replication and virus yield at 2 and 4 days postchallenge, in comparison with the high levels of SARS-CoV-2 genomic RNA and infectious virus detected in control infected mice or in mice immunized with one dose of MVA-CoV2-S (Fig. 1b, c).

The histopathological evaluation of the lung showed that mice vaccinated with two doses of MVA-CoV2-S displayed at 4 days postchallenge significantly lower lung lesion scores (Fig. 1d, left panel and Supplementary Fig. 1a) and lower percentages of lung area with lesions (Fig. 1d, right panel) than control MVA-WT inoculated mice or mice immunized with one dose of MVA-CoV2-S, which could be indicative of low breakthrough infections. Mice vaccinated with two doses of MVA-CoV2-S only displayed focal thickening of the alveolar septae, and occasional presence of inflammatory cells within the alveoli (Supplementary Fig. 1b). However, mice immunized with one dose of MVA-CoV2-S or control MVA-WT inoculated mice showed more severe diffuse thickening of the alveolar septae, higher presence of mononuclear cell infiltrates within alveolar spaces, as well as the presence of larger multifocal perivascular and peribronchiolar mononuclear infiltrates. Unvaccinated mock-infected mice did not display remarkable inflammatory lesions (Supplementary Fig. 1b).

Next, we evaluated the effect of MVA-CoV2-S on the cytokine expression pattern induced in mice, as an extensive upregulation of several pro-inflammatory cytokines are associated with COVID-19 disease progression and severity^17–19^. Thus, at days 2 and 4 postchallenge, mRNA levels of key cytokines on lung homogenates were analyzed by RT-qPCR and the results showed that two doses of MVA-CoV2-S significantly down-regulated IL-6, TNF-α, IL-10, IP-10, and IFN-γ mRNA levels, compared to control infected mice or mice immunized with one dose of MVA-CoV2-S (Fig. 1e).

### MVA-CoV2-S triggered in K18-hACE2 transgenic mice high titers of S- and RBD-specific IgG antibodies that neutralized different SARS-CoV-2 variants of concern (VoC)

We next evaluated SARS-CoV-2-specific humoral responses induced in K18-hACE2 transgenic mice vaccinated with one or two doses of MVA-CoV2-S.

**Fig. 2:**
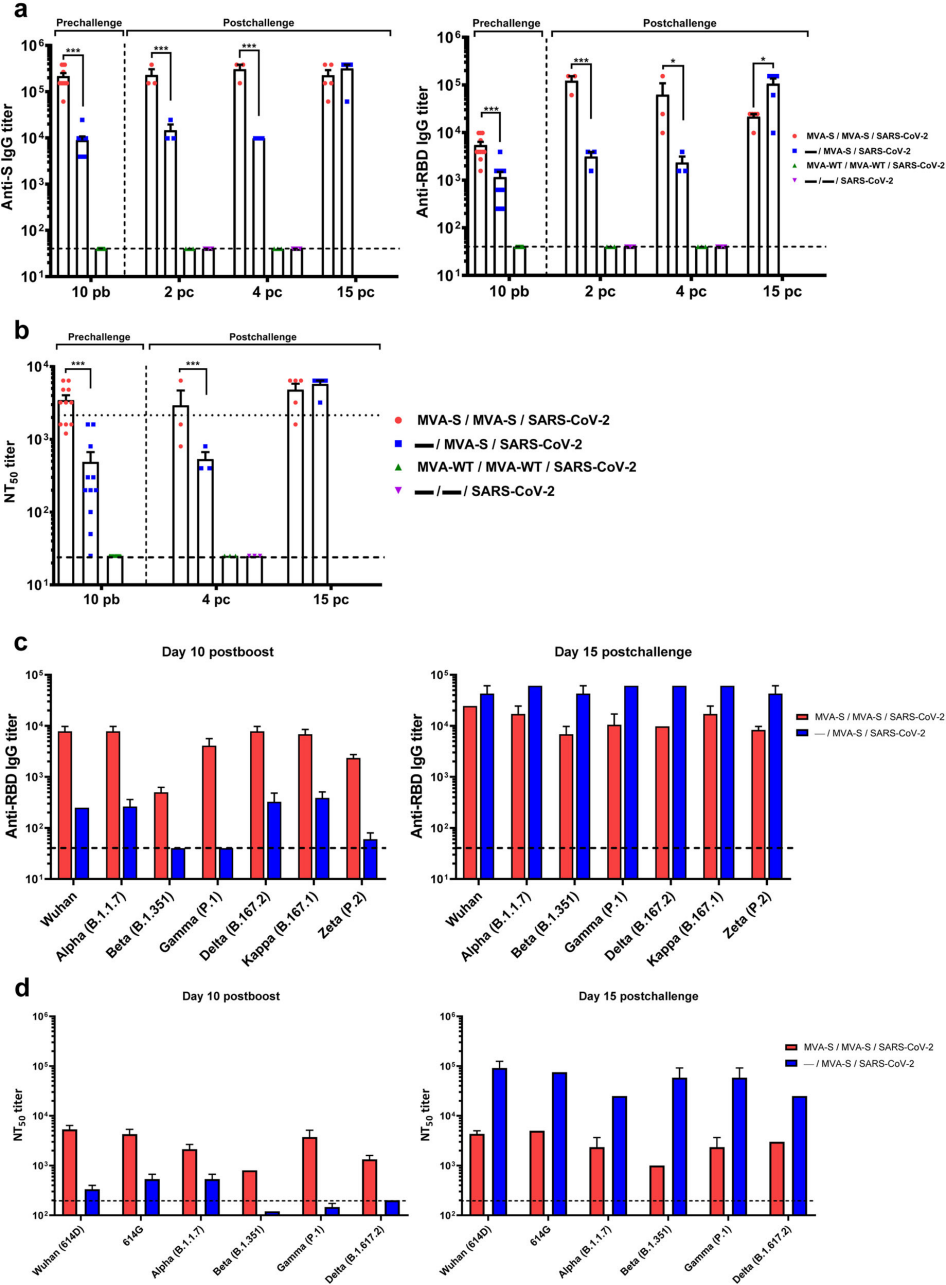
MVA-CoV2-S vaccine candidate induced high levels of humoral responses in vaccinated and challenged K18-hACE2 transgenic mice. **a** Titers of IgG antibodies specific for the S (left) and RBD (right) proteins (Wuhan strain). Determined by ELISA in individual mouse serum samples collected at day 10 post-boost (prechallenge; *n* = 11/group) and at days 2 (*n* = 3/group), 4 (*n* = 3/group) and 15 (*n* = 5/group) postchallenge from K18-hACE2 mice. Mean values and SEM are represented. Dashed line represents the limit of detection. **b** SARS-CoV-2 neutralizing antibody titers. NT_50_ titers were evaluated in individual mouse serum samples collected at day 10 post-boost (prechallenge) and at 4 and 15 days postchallenge, using retrovirus-based pseudoparticles expressing the SARS-CoV-2 S protein (Wuhan strain). Mean NT_50_ values and SEM are represented. Upper dotted line represents the levels obtained with the NIBSC 20/136 international standard plasma (containing pooled plasma obtained from 11 individuals recovered from SARS-CoV-2 infection). Bottom dotted line represented the limit of detection. **c** Titers of IgG antibodies against RBD from different SARS-CoV-2 VoC. Determined by ELISA in pooled sera samples collected at days 10 post-boost (left) and 15 postchallenge (right). **d** SARS-CoV-2 neutralizing antibody titers against SARS-CoV-2 VoC. NT_50_ titers were evaluated in pooled mouse serum samples collected at days 10 post-boost (left) and 15 postchallenge (right), using VSV-based pseudoparticles expressing the SARS-CoV-2 S protein of different VoC. Mean NT_50_ values and SEM are represented. Student’s *t*-test: *P < 0.05; ***P < 0.001.

K18-hACE2 mice immunized with MVA-CoV2-S elicited high titers of S- and RBD-specific IgG antibodies at 10 days post-boost, although the two-dose regimen induced about 25 times higher titers than one-dose treatment (Fig. 2a). Mice vaccinated with two MVA-CoV2-S doses induced similar anti-S and anti-RBD IgG titers at prechallenge, and at 2, 4, and 15 days postchallenge; in contrast mice immunized with one dose of MVA-CoV2-S developed a robust humoral immunity against SARS-CoV-2 associated, in part, with a breakthrough infection between days 4 and 15 postchallenge (Fig. 2a). Consistently, the serum of animals immunized with two doses of MVA-CoV2-S better neutralized S-pseudotyped retroviruses (Fig. 2b) and SARS-CoV-2 live virus (Supplementary Fig. 2), with titers superior to those of an anti-SARS-CoV-2 human immunoglobulin WHO international standard (NIBSC 20/136) (Fig. 2b). However, the neutralization titers in mice that received both immunization regimes were similar at 15 days after SARS-CoV-2 infection (Fig. 2b).

Analysis of IgG isotypes against S and RBD proteins at the prechallenge and postchallenge timepoints showed higher titers of IgG2c than IgG1 antibodies in both immunization regimens, leading to a IgG2c/IgG1 ratio higher than 1 (Table 1), which is indicative of a Th1-type protective humoral response^20^.

**Table 1:** Isotype analysis of anti-S and anti-RBD IgG antibodies in immunized mice.

Timepoints analyzed	IgG2c and IgG1 titers and Ratio IgG2c/IgG1 ^a^																	
	MVA-S / MVA-S / SARS-CoV-2						— / MVA-S / SARS-CoV-2						MVA-S / MVA-S					
	(K18-hACE2 mice)						(K18-hACE2 mice)						(C57BL/6 mice)					
	S			RBD			S			RBD			S			RBD		
	IgG1	IgG2c	IgG2c/IgG1	IgG1	IgG2c	IgG2c/IgG1	IgG1	IgG2c	IgG2c/IgG1	IgG1	IgG2c	IgG2c/IgG1	IgG1	IgG2c	IgG2c/IgG1	IgG1	IgG2c	IgG2c/IgG1
10 days post-boost (prechallenge)	24.414	152.587	6,25	1.562	61.035	39,07	1.562	9.765	6,25	625	3.906	6,25	–	–	–	–	–	–
Day 4 postchallenge	24.414	152.587	6,25	438	61.035	139,51	1.562	9.765	6,25	100	3.906	39,06	–	–	–	–	–	–
Day 15 postchallenge	24.414	152.587	6,25	1.562	61.035	39,07	152.587	953.674	6,25	9.765	152.587	15,63	–	–	–	–	–	–
Day 6 post-rechallenge	24.414	152.587	6,25	625	42.724	69,36	61.035	953.674	15,63	2.734	152.587	55,81	–	–	–	–	–	–
6 months post-boost	–	–	–	–	–	–	–	–	–	–	–	–	3.906	17.089	4,38	625	9.765	15,62

^a^Mean titers of IgG2c and IgG1 isotype antibodies against S and RBD proteins from duplicates of pooled sera samples obtained from the different immunization regimens studied are represented, including the ratio IgG2c/IgG1. –: not applicable.

Remarkably, at day 10 post-boost IgG titers against RBD proteins from different SARS-CoV-2 VoC were similarly high after two MVA-CoV2-S doses, except for the beta (B.1.351) variant, which was about 15 times lower (Fig. 2c, left). After a single dose, we observed lower IgG titers against the beta, gamma (P.1), and zeta (P.2) variants that contained mutations at the RBD E484 residue. Nonetheless, the IgG titers against all VoC were comparable after mice infection with SARS-CoV-2 (Fig. 2c, right), but the one-dose regimen induced higher titers as a result of a breakthrough infection (Fig. 2c, right). The VoC neutralization titers followed similar trend as the anti-RBD IgG antibodies, as they are higher in serum of mice immunized with two MVA-CoV2-S doses, being again the beta variant the less recognized VoC (Fig. 2d, left); in mice serum samples collected after one vaccine dose the beta, gamma, and delta (B.167.2) variants were poorly neutralized (Fig. 2d, left). SARS-CoV-2 infection markedly boosted neutralization titers against all VoC in single-dose immunized mice, whereas the neutralizing titers against all VoC remained almost unchanged in mice that received two doses (Fig. 2d, right).

### Binding and neutralizing antibodies correlate with protection

**Fig. 3:**
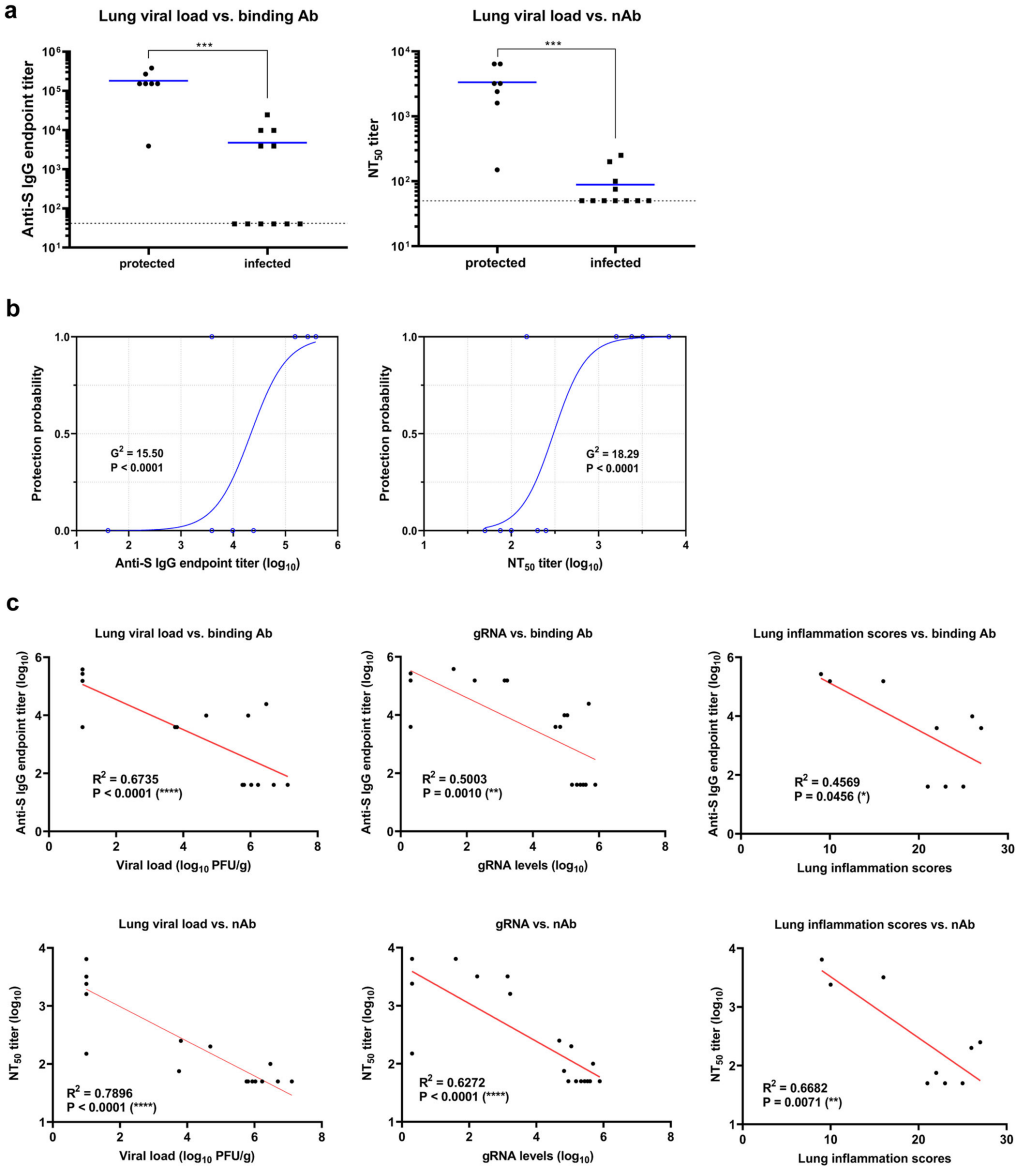
Binding IgG and neutralizing antibodies correlate with protection. **a** K18-hACE transgenic mice were immunized with one or two doses of MVA-CoV2-S, or with MVA-WT, and then inoculated with 1 × 10^5^ PFU of SARS-CoV-2, and 2 and 4 days later sacrificed for virological analysis of lung tissue (*n* = 18). Prior to virus inoculation (at 10 days post-boost) serum samples were analyzed for anti-S IgG antibody titers and virus-neutralizing antibody titers. Protection was defined as a viral load below 5 × 10^2^ PFU/g in lung tissue, irrespective of vaccine regimen (Fig. 1c). Median antibody responses per group is indicated with horizontal lines. Dotted lines indicate the limit of detection. **b** Logistic regression models were built with binding anti-S and neutralizing antibody titers from pooled data as an independent variable, and protection outcome as the dependent variable. **c** Correlation analysis. Correlation of binding anti-S IgG endpoint titers (upper row) and NT_50_ titers (bottom row) (in log10) determined 3.5 weeks before challenge (10 days post-boost) with viral load (log10 PFU/g in lung tissue) (left), genomic RNA levels (log10 genomic RNA in A.U,) (middle) and lung inflammation scores (log10) (right) determined at 2 and 4 days postchallenge. Red lines reflect the best linear fit relationship between these variables. *R*^2^ and *P*-values reflect two-sided Pearson correlation coefficients.

Next, we tried to correlate antibody titers with infection outcomes. Thus, anti-S IgG antibody and neutralizing titers from sera collected at day 10 post-boost (prechallenge) from protected and non-protected vaccinated K18-hACE2 transgenic mice were compared, in order to identify correlates of protection (Fig. 3a). SARS-CoV-2-infected mice with a lung viral load below 5 × 10^2^ PFU/g lung tissue were considered as protected (see Fig. 1c). Protected mice immunized with MVA-CoV2-S elicited 37-38 fold higher anti-S and neutralizing antibody titers than non-protected mice (Fig. 3a). Additionally, we studied the relationship of antibody response levels and protection outcome using a simple logistic regression model, which showed that antibody titers above 10^4.5^ and 10^2.5^ for anti-S and neutralization, respectively, protected from lung infection in 85.7% of the animals (Fig. 3b).

To further understand the correlation between humoral immune responses and protection, we calculated the Pearson correlation coefficient to analyze the relationship of antibody response levels with parameters such as viral load, genomic RNA levels, and lung inflammation scores (Fig. 3c). Both anti-S IgG antibody endpoint titers and live-virus NT_50_ titers correlated inversely with lung viral load, genomic RNA levels, and lung inflammation scores. In general, neutralizing antibody titers correlated better with those protection parameters than anti-S IgG titers, with a strong negative correlation between neutralizing antibodies titers and lung viral load (*R*_2_ = 0.7896; *p* < 0.0001).

### K18-hACE2 transgenic mice vaccinated with one and two doses of MVA-CoV2-S are protected against SARS-CoV-2 reinfection

**Fig. 4:**
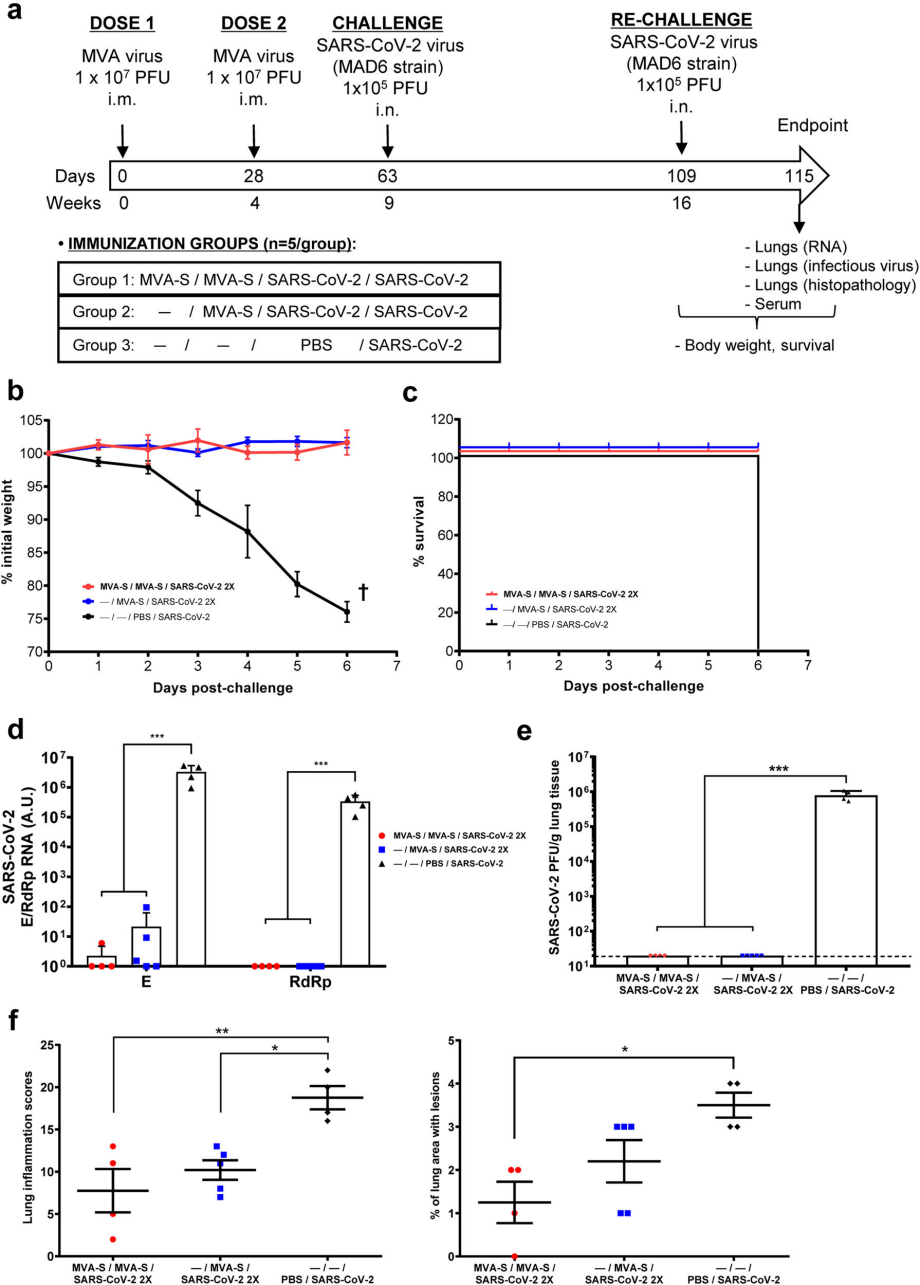
Outcome of SARS-CoV-2 inoculation (rechallenge) in immunized K18-hACE2 mice that recovered from first virus infection. **a** Scheme of immunization and SARS-CoV-2 infection procedures in K18-hACE2 mice (*n* = 5 per group), to determine the effect of second virus inoculation. Body weight (**b**) and mortality (**c**) were monitored for 6 days after rechallenge with SARS-CoV-2 virus. All non-vaccinated mice (group 3) died at day 6 post-rechallenge. SARS-CoV-2 genomic (RdRp gene) and subgenomic (E gene) RNA (**d**) and infectious virus (**e**) in lung samples at day 6 postchallenge were evaluated as indicated in Fig. 1. **f** Lung inflammation scores and percentage of lung area with lesions, in lung samples at day 6 post-rechallenge. Mean and SEM of cumulative histopathological lesion scores (left) and percentage of lung area affected by inflammatory lesions (right). Student’s *t*-test: *P < 0.05; **P < 0.005; ***P < 0.001.

Next, we evaluated whether K18-hACE2 mice immunized with one or two doses of MVA-CoV2-S (*n* = 5/group) were protected against a SARS-CoV-2 reinfection performed 7 weeks after the first SARS-CoV-2 challenge (Fig. 4a); body weight and mortality were monitored daily before animals were sacrificed at 6 days post-rechallenge.

All mice vaccinated with one or two doses of MVA-CoV2-S did not lose body weight (Fig. 4b) and survived (Fig. 4c), while all unvaccinated and challenged mice lost body weight and died at 6 days after rechallenge (Fig. 4b, c). The analysis of SARS-CoV-2 virus replication in lung samples at day 6 post-reinfection showed that both MVA-CoV2-S immunization regimes completely abrogated SARS-CoV-2 replication (subgenomic and genomic mRNA) (Fig. 4d) and virus yields (Fig. 4e). Furthermore, both groups of vaccinated mice displayed lower lung lesion scores and percentages of damaged lung area than control infected mice (Fig. 4f and Supplementary Fig. 3a, b).

**Fig. 5:**
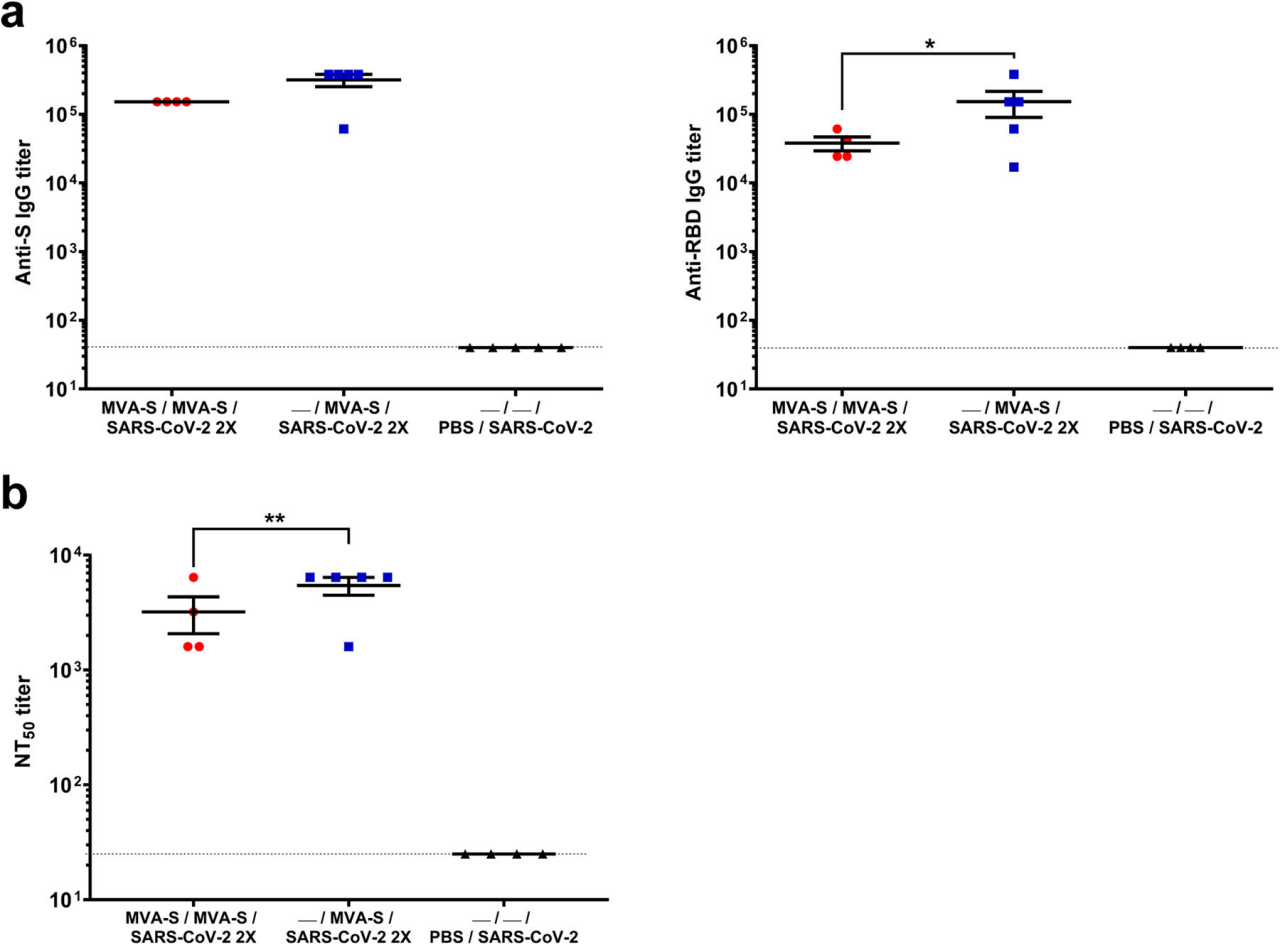
Anti-S and -RBD antibodies that neutralized SARS-CoV-2 after SARS-CoV-2 rechallenge in immunized K18-hACE2 mice that recovered from first virus infection. **a** Titers of IgG antibodies specific for the S (left) and RBD (right) proteins. Determined by ELISA as in Fig. 2a. **b** SARS-CoV-2 neutralizing antibody titers. Determined by using retrovirus-based pseudoparticles expressing the SARS-CoV-2 S protein (Wuhan strain) as in Fig. 2b. Individual mouse serum samples collected 6 days after rechallenge were analyzed. Student’s *t*-test: *P < 0.05; **P < 0.005.

Evaluation of SARS-CoV-2-specific humoral responses at day 6 post-reinfection showed that vaccinated mice elicited high titers of S- and RBD-specific IgG antibodies. Mice that received a single MVA-CoV2-S dose had slightly higher levels of anti-S IgG antibodies and significantly higher anti-RBD IgG titers than mice vaccinated with two doses (Fig. 5a). Furthermore, vaccinated mice induced S- and RBD-specific IgG2c/IgG1 ratios higher than 1 (Table 1), indicative again of a Th1-type response. Moreover, immunized mice elicited high titers of SARS-CoV-2 neutralizing antibodies, again with the one-dose treatment triggering significant higher neutralizing titers than the two-dose regimen (Fig. 5b).

### Immunization of C57BL/6 mice with two doses of MVA-CoV2-S induces long-term SARS-CoV-2 S-specific CD4^+^ and CD8^+^ T-cell and humoral memory immune responses

We previously described that MVA-CoV2-S induced potent adaptive S-specific CD4^+^ and CD8^+^ T-cell and humoral immune responses^5^, but the longevity of these responses was unknown. Thus, groups of C57BL/6 mice (*n* = 5/group) were immunized twice with MVA-CoV2-S (intramuscularly, 1 × 10^7^ PFU/mouse), at weeks 0 and 2, and 6 months later animals were sacrificed to evaluate the long-term SARS-CoV-2-specific T-cell and humoral immune responses induced in splenocytes and serum samples, respectively.

**Fig. 6:**
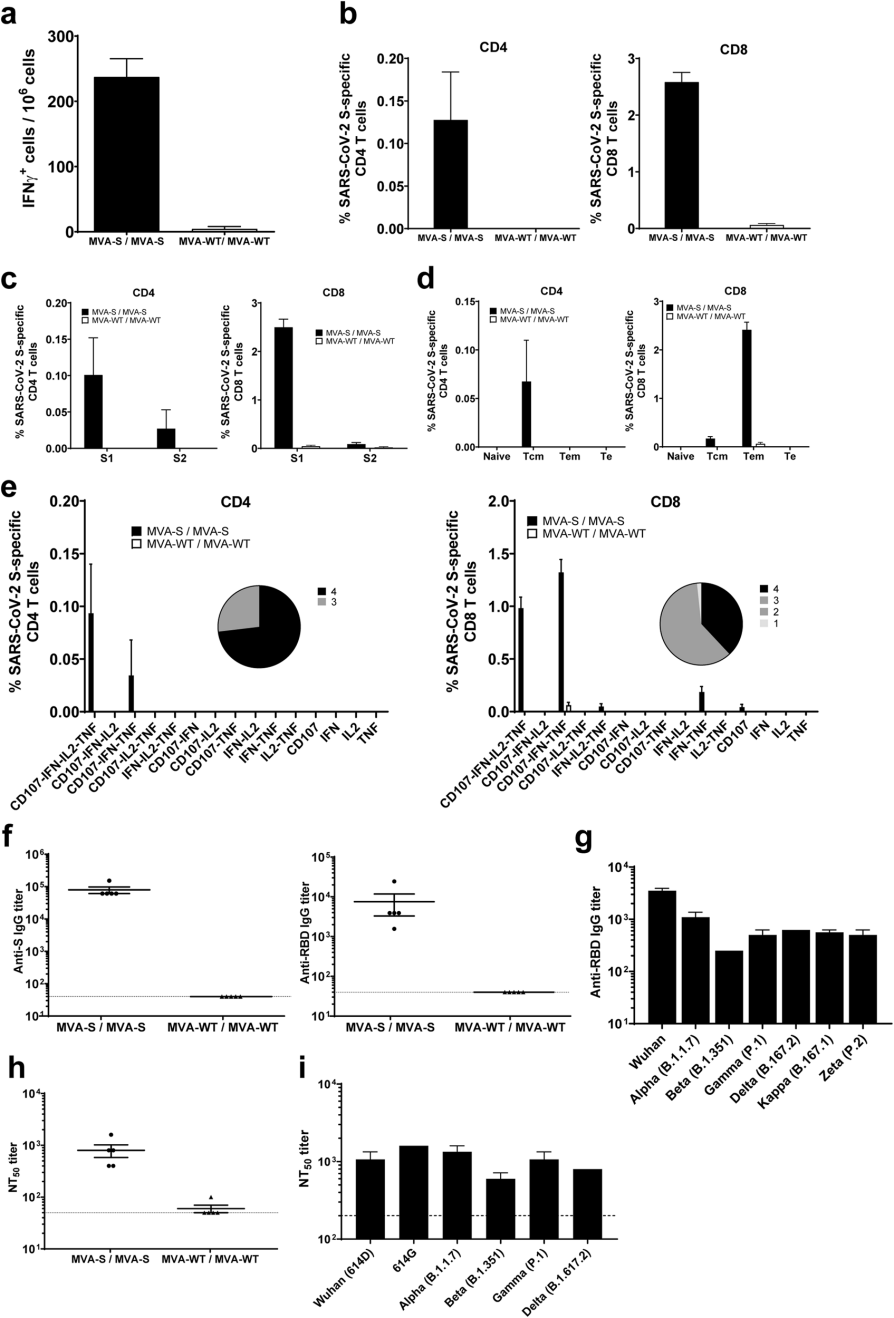
MVA-CoV2-S elicited long-lasting SARS-CoV-2 S-specific T-cell and humoral immune responses in immunized C57BL/6 mice. **a** Magnitude of SARS-CoV-2 S-specific cell responses. Cells secreting IFN-γ per million of splenocytes and directed against S1 + S2 peptide pools in immunized C57BL/6 mice were evaluated at 6 months post-boost by an ELISpot assay from a pool of splenocytes derived from 5 immunized mice per group. Mean values and standard deviation of triplicate samples. **b** Magnitude of S-specific CD4^+^ and CD8^+^ T-cell immune responses evaluated at 6 months post-immunization. Percentages of CD4^+^ or CD8^+^ T cells expressing CD107a and/or producing IFN-γ and/or TNF-α and/or IL-2 against a mixture of S1 and S2 peptide pools in immunized mice. Cell percentages were determined by ICS from splenocyte pools. **c** S-specific T-cell immune responses against S1 and S2 regions. Percentages of S1- or S2-specific CD4^+^ and CD8^+^ T cells determined as in panel B. **d** Percentages of Naïve (CD127^-^/CD62L^−^), T central memory (Tcm, CD127^+^/CD62L^+^), T effector memory (Tem, CD127^+^/CD62L^−^), and T effector (Te, CD127^−^/CD62L^−^) CD4^+^ and CD8^+^ T cells specific for S1 and S2 peptide pools, and expressing any of the markers CD107a, IFN-γ, TNF-α, and IL-2. **e** Polyfunctional profiles (based on the expression of selected markers CD107a, IFN-γ, TNF-α, and IL-2) of total S-specific CD4^+^ or CD8^+^ T-cell immune responses directed against a mixture of S1 and S2 peptide pools. The pie charts summarize the percentage of S-specific T cells exhibiting 1, 2, 3, or 4 markers. **f** Titers of IgG antibodies specific for the S (left) and RBD (right) proteins. Determined by ELISA in individual serum samples as in Fig. 2a. **g** Titers of IgG antibodies against RBD from different SARS-CoV-2 VoC. Determined by ELISA in pooled sera samples as in Fig. 2c. **h** SARS-CoV-2 neutralizing antibody titers. Determined by using retrovirus-based pseudoparticles expressing the SARS-CoV-2 S protein (Wuhan strain) in individual serum samples as in Fig. 2b. **i** SARS-CoV-2 neutralizing antibody titers against SARS-CoV-2 VoC. NT_50_ titers were evaluated by using VSV-based pseudoparticles expressing the SARS-CoV-2 S protein of different VoC, as in Fig. 2d. Individual or pooled mouse serum samples collected at 6 months post-boost were analyzed.

Mice vaccinated with two doses of MVA-CoV2-S elicited high levels of long-term IFNγ-secreting cells, reactive to a mixture of SARS-CoV-2 S1 + S2 peptide pools, as revealed by ELISpot (Fig. 6a). Robust long-term S-specific CD4^+^ and CD8^+^ T-cells expressing CD107a, and secreting IFNγ, TNFα, and/or IL-2 were induced, with a higher overall response mainly mediated by the CD8^+^ T-cell repertoire and a CD4^+^ Th1 profile, as measured by intracellular cytokine staining (ICS) (Fig. 6b). Long-term S-specific CD4^+^ and CD8^+^ T-cell responses were directed mainly against the S1 peptide pool (Fig. 6c). S-specific CD4^+^ and CD8^+^ T-cell responses were mostly of the T central memory and T effector memory phenotypes, respectively (Fig. 6d). Moreover, the analysis of the quality of the S-specific T-cell responses showed that MVA-CoV2-S triggered highly polyfunctional long-term CD4^+^ and CD8^+^ T cells (Fig. 6e), with 100 and 90% of S-specific CD4^+^ and CD8^+^ T cells, respectively, exhibiting three or four functions (Fig. 6e, pie charts). In particular, CD4^+^ T cells expressing CD107a-IFNγ-TNFα-IL-2 and CD8^+^ T cells expressing CD107a-IFNγ-TNFα were the most abundant CD4^+^ and CD8^+^ T cell populations (Fig. 6e, bars).

The analysis of SARS-CoV-2-specific humoral immune responses showed that mice vaccinated with two doses of MVA-CoV2-S induced long-term high titers of S- and RBD-specific binding IgG antibodies (Fig. 6f). Induced IgG antibodies also recognized RBD proteins from several VoC, although IgG titers to the beta variant were lower than to the other VoC (Fig. 6g). Furthermore, S- and RBD-specific IgG2c/IgG1 ratios were above one (Table 1), likely suggestive of a Th1-like immune response. Remarkably, high titers of SARS-CoV-2 neutralizing antibodies were maintained at 6 months post-immunization with MVA-CoV2-S (Fig. 6h) and recognized several VoC, being the beta variant the less recognized VoC (Fig. 6i). The extrapolation of neutralizing antibody titers induced at 6 months post-immunization in C57BL/6 mice to the regression model performed in protected and non-protected K18-hACE2 mice (Fig. 3a, b) suggested that long-lived antibody levels induced should have over 90% probability to confer protection.

## Discussion

Despite the wide variety of vaccine candidates against COVID-19 with robust immunogenicity and efficacy, there is still a global high-priority for a stable, safer, more immunogenic, and effective COVID-19 vaccine against all kinds of emerging variants. Moreover, it remains to be defined the durability of the immune responses and efficacy in the long run. One promising vaccine vector is the highly attenuated poxvirus strain MVA, non-replicative in human cells that have been extensively studying in preclinical and clinical trials as vaccine candidate against a wide spectrum of diseases with excellent safety, immunogenicity, and efficacy profiles^21,22^.

Recently, we described the generation and immunogenic characterization of the MVA-CoV2-S vaccine candidate against COVID-19, which is based on an MVA vector that expresses a human codon optimized full-length SARS-CoV-2 S protein that was highly immunogenic in mice, inducing a robust activation of both arms of the adaptive immune system, T and B cells^5^. Furthermore, we also reported that one or two doses of MVA-CoV2-S controlled morbidity (weight loss) and mortality caused by SARS-CoV-2 infection in K18-hACE2 transgenic mice^5^, a SARS-CoV-2 susceptible mouse model that exhibits fatal respiratory infection and mortality following intranasal SARS-CoV-2 administration^13–16^. Here, we provide a detailed evaluation of the efficacy and immunogenicity of one or two doses of MVA-CoV2-S in K18-hACE2 transgenic mice. The results obtained indicated that two doses of MVA-CoV2-S resulted in the highest level of protection. At 2 and 4 days, postchallenge SARS-CoV-2 replication in the lungs (genomic mRNA and viral load) was completely abolished, and histopathological lung lesions and levels of pro-inflammatory cytokines were significantly reduced when compared to control infected mice or mice vaccinated with one dose of MVA-CoV2-S. Since SARS-CoV-2 replication in the lungs was completely abolished in mice vaccinated with two doses of MVA-CoV2-S, it is likely that virus shedding in these animals is limited, because it has been reported that SARS-CoV-2 shedding in transgenic mice is often associated with a high viral titer in lung tissues and vaccinated transgenic mice did not shed virus as early as day 2 postinfection, when virus was cleared from the lungs^6^. Although a single dose of MVA-CoV2-S had a significant inhibitory effect in RNA and viral load at 4 days postchallenge, other parameters analyzed indicated that one dose does not completely protects from lung infection, at least in the first days postinfection.

A detailed analysis of histopathological lung lesions revealed that after challenge with SARS-CoV-2, mice vaccinated with two doses of MVA-CoV2-S displayed less extensive and less severe lung inflammatory lesions compared to mice immunized with one dose of MVA-CoV2-S or control infected mice. The most severely affected mice displayed inflammatory lesions similar to those described in previous experimental infections with SARS-CoV-2 in transgenic ACE2 mice^23^ and non-human primates^24^, and resembled histopathological lung lesions observed in human patients infected with SARS-CoV-2^25,26^. Moreover, surviving K18-hACE2 mice immunized with one or two doses of MVA-CoV2-S and challenged with a second lethal dose of SARS-CoV-2 displayed significantly lower lung lesions scores compared to control infected mice. Some mice, especially those immunized with two doses of MVA-CoV2-S, did not display focal lesions.

Overproduction of several pro-inflammatory cytokines is a marker of COVID-19 disease progression and mortality^17–19,27–29^. Here, we found that after SARS-CoV-2 infection of transgenic K18-hACE2 mice vaccinated with two doses of MVA-CoV2-S there was a significant downregulation in the mRNA levels of several pro-inflammatory cytokines in lung homogenates, such as IL-6, TNF-α, IL-10, IFN-γ, and IP-10, compared to control infected mice where high levels of these cytokines were detected. All these results indicated that two doses of MVA-CoV2-S prevented the increase in pro-inflammatory cytokines induced by SARS-CoV-2 infection, helping to prevent COVID-19 related lung damage. Other MVA-based vaccine candidates against SARS-CoV-2 have probe to reduce transcripts associated with inflammation and hyperimmune activation in lungs of vaccinated and challenged mice^6^ and non-human primates^7^.

Of significance, MVA-CoV2-S induced high titers of binding IgG antibodies against S and RBD and neutralizing antibodies against SARS-CoV-2, with the two-dose regime eliciting higher titers than the one-dose regime. Remarkably, MVA-CoV2-S vaccination also induced high titers of IgG and neutralizing antibodies recognizing several VoC (alpha, beta, gamma, delta, kappa, and zeta), being beta the less recognized variant. This highlights the potent and wide spectrum of humoral responses elicited by MVA-CoV-2 vaccine candidates able to neutralize different VoC, especially the delta VoC that is actually the main strain circulating in the human population. Additionally, a Th1-type immune response was induced in all vaccinated mice. All those specific immune markers have been linked to SARS-CoV-2 vaccine efficacy and control of COVID-19 progression, such as high titers of IgG antibodies against SARS-CoV-2 S and RBD proteins^30^, a Th1 profile immune response^20^, induction of potent SARS-CoV-2 neutralizing antibodies^31,32^, and low levels of pro-inflammatory cytokines^19^. Furthermore, evidence from preclinical studies in non-human primates, hamsters, and transgenic mice indicates that vaccine-induced SARS-CoV-2 neutralizing antibodies correlate with protection against lung infection and clinical disease^33–36^. Moreover, a longitudinal study of SARS-CoV-2-infected patients also reveals a high correlation between neutralizing antibodies and COVID-19 severity^37^. Here, the robust protection observed in K18-hACE2 transgenic mice vaccinated with two doses of MVA-CoV2-S (determined by the low levels of SARS-CoV-2 RNA, viral load, and inflammation scores in lung samples) significantly correlated with high titers of binding IgG and neutralizing antibodies; although neutralizing antibody titers correlated better with protection than binding anti-S IgG antibody titers. The NT_50_ titers elicited by two doses of MVA-CoV2-S were higher than 3 × 10^3^, and were comparable to those induced by other viral vector-based vaccines, such as ChAdOx1 nCoV-19^38^, and different recombinant MVA vectors also expressing the full-length S protein^4,6–8^, or higher than those of an anti-SARS-CoV-2 human immunoglobulin WHO international standard, reflecting the potent humoral responses induced by the vaccine candidate. Furthermore, we also found a strong correlation between the neutralizing antibody NT_50_ titers measured using live SARS-CoV-2 or retrovirus-based pseudoparticles expressing the S protein, similarly as previously reported^37^.

Here, we also found that either one or two doses of MVA-CoV2-S induced a predominantly Th1-type response to S and RBD proteins, with IgG2c > IgG1. This is relevant, as activation of a strong Th1 cell response has been associated with less severe cases of COVID-19, while Th2 cell responses have been associated with more severe lung disease in humans^39^. Moreover, previous studies also demonstrated the induction of predominantly Th1-type immune responses in mice immunized with MVA-based vaccines encoding SARS-CoV-2 S antigens^6,8^ or by other COVID-19 vaccines^20^.

Mice vaccinated with two doses of MVA-CoV2-S induced similar high titers of IgG and neutralizing antibodies against parental SARS-CoV-2 and VoC before and after SARS-CoV-2 infection. However, at later timepoints after SARS-CoV-2 infection animals vaccinated with one dose of MVA-CoV2-S developed an anamnestic response through breakthrough infection, with higher levels of S- and RBD-specific IgGs and neutralizing antibodies against parental SARS-CoV-2 and VoC at 15 days postchallenge that were similar or superior to those elicited by the two-dose regime. This indicates that SARS-CoV-2 infection boosted the immune system and induced comparable humoral responses to two MVA-CoV2-S doses.

All vaccinated animals were completely resistant to a second SARS-CoV-2 reinfection 7 weeks after the first challenge, independently of the one or two dose vaccination regimes. The high levels of SARS-CoV-2-specific antibodies induced at late timepoints after infection with both immunization regimes were sufficient to confer protection against a reinfection. Therefore, vaccinated individuals with two MVA-CoV2-S doses and infected people that had received a single vaccine dose should, in principle, be protected from a first or second infection, respectively, as shown here with animal models.

Generation of robust, durable and Th1-skewed T- and B-cell memory immune responses are crucial to prevent SARS-CoV-2 infection, to stop virus replication and to facilitate virus clearance without respiratory complications^40^, which is the main goal of SARS-CoV-2 vaccines. Recently, it has been reported the persistence of antibodies 6 months after the second dose of mRNA-1273 vaccine (Moderna) in a phase 1 follow-up study^41^. Moreover, SARS-CoV-2-specific T and B cell responses during COVID-19 disease progression have been tracked in blood samples, and individuals recovered from mild COVID-19 develop and sustain multifaceted SARS-CoV-2-specific immunological memory: SARS-CoV-2-specific IgG antibodies, neutralizing plasma, and memory B and T cells that persisted for at least 3 months^42^. Of significance, our results demonstrated that, similar to natural infection and to other vaccines administered in the human population, immunization of C57BL/6 mice with two doses of MVA-CoV2-S vaccine candidate induces long-term, strong, and polyfunctional SARS-CoV-2 S-specific CD4^+^ and CD8^+^ T-cell memory immune responses, as well as long-term SARS-CoV-2-specific humoral immune responses (binding IgG and neutralizing antibodies) against parental Wuhan strain and several VoC. Those responses were detected at 6 months after the last immunization, with titers of neutralizing antibodies that are presumed to correlate with protection. Notably, all the animals remained healthy after 6 months of follow-up, suggesting safety with no adverse effects of the MVA-CoV2-S vaccine.

All the efficacy and immunogenicity results reported here support that two doses of MVA-CoV2-S induced robust protection in transgenic K18-hACE2 mice, with the one-dose regimen being less effective. Other groups have also recently reported the generation of MVA vectors expressing SARS-CoV-2 S antigens that elicited efficacy in susceptible mice and macaques with one or two doses^4,6–8^, similar to the reported in this study with transgenic K18-hACE2 mice. In particular, Liu and colleagues have also shown control of SARS-CoV-2 infection by a MVA vector vaccine in upper and lower respiratory tracts, a critical event to prevent SARS-CoV-2 transmission^6^.

Will the poxvirus vectors have a niche in the current market with several established and efficacious vaccines against SARS-CoV-2? We still do not know the durability of the S-specific immune responses, and booster doses are now considered to assure long-term and broader efficacy. Poxvirus vectors enhance immune responses when used in combination with other vaccine technologies, being most effective when delivered as a booster, such as we and others described^5,11,43,44^. Hence, vaccines based on MVA or other poxvirus vectors could be implemented in clinical trials, either alone or in combination with other vaccines currently in the market, and provide an advantage in the general control of the COVID-19 pandemic. The MVA-CoV2-S dose used in this mouse preclinical study (1 × 10^7^ PFU/mouse) corresponds to a low human dose, as the standard dose for MVA-based vaccines used in clinical trials is 1 × 10^8^ PFU/human.

In summary, the present study provided valuable evidence of MVA-CoV2-S preclinical efficacy and immunogenicity, adding further support for the evaluation of this vaccine in clinical trials.

## Methods

### Ethics statement

Female transgenic K18-hACE2 mice, expressing the human angiotensin-converting enzyme-2 (ACE2) receptor, were obtained from The Jackson Laboratory (034860-B6.Cg-Tg(K18-ACE2)2Prlmn/J, genetic background C57BL/6 J × SJL/J)F2) and efficacy experiments were performed in the biosafety level 3 (BSL-3) facilities at Centro de Investigación en Sanidad Animal (CISA)-Instituto Nacional de Investigaciones Agrarias (INIA-CSIC) (Valdeolmos, Madrid, Spain). Female C57BL/6OlaHsd mice (6–8 weeks old) used for long-term immunogenicity assays were purchased from Envigo Laboratories and stored in the animal facility of Centro Nacional de Biotecnología (CNB) (Madrid, Spain). The efficacy and immunogenicity animal studies were approved by the Ethical Committee of Animal Experimentation (CEEA) of the CNB (Madrid, Spain) and by the Division of Animal Protection of the Comunidad de Madrid (PROEX 49/20, 169.4/20 and 161.5/20). Animal procedures conformed with international guidelines and with Spanish law under the Royal Decree (RD 53/2013).

### Viruses

The poxviruses used in this study included the attenuated MVA-WT strain obtained from the Chorioallantois vaccinia virus Ankara (CVA) strain after 586 serial passages in CEF cells^45^, and the MVA-CoV2-S vaccine candidate expressing a human codon optimized full-length SARS-CoV-2 S protein^5^.

SARS-CoV-2 strain MAD6 (kindly provided by José M. Honrubia and Luis Enjuanes, CNB-CSIC, Madrid, Spain) is a virus collected from a nasopharyngeal swab from a 69-year-old male COVID-19 patient from Hospital 12 de Octubre in Madrid^46^. The stock virus was prepared by collecting the supernatant from Vero-E6 cells (ATCC catalog no. CRL-1586), and was isolated, plaque cloned three times, and amplified by propagation in Vero-E6 cells by inoculation at a multiplicity of infection (MOI) of 0.001 PFU/cell (passage 2). Cell supernatants were harvested at 72 h postinfection, cleared by centrifugation, aliquoted, and stored at −80 °C. Virus infectivity titers were determined by standard plaque or median tissue culture infectious dose (TCID50) assays in Vero-E6 cells. Full-length virus genome was sequenced, and it was found to be identical to SARS-CoV-2 reference sequence (Wuhan-Hu-1 isolate, GenBank: MN908947), except the silent mutation C3037 > T, and two mutations leading to amino acid changes: C14408 > T (in nsp12) and A23403 > G (D614G in S protein).

### Efficacy study schedule in K18-hACE2 transgenic mice

Female K18-hACE2 mice (10 weeks old at the beginning of the study) immunized with one or two doses of MVA-CoV2-S were used to evaluate the efficacy of the MVA-CoV2-S vaccine candidate. Groups of animals (*n* = 11) received one or two doses of 1 × 10^7^ PFU of MVA-CoV2-S by intramuscular route in 100 μl of PBS (50 μl/leg) at 0 and 4 weeks. Mice primed and boosted with nonrecombinant MVA-WT or not vaccinated were used as control groups. At week 9, mice were challenged with a lethal dose (1 × 10^5^ PFU) of SARS-CoV-2 (MAD6 strain) by intranasal route in 50 μl of PBS, and one group of unvaccinated mice were left uninfected. Mice were monitored for body weight change and mortality for 15 days postchallenge. Animals with more than 25% of weight loss were euthanized. At days 2 and 4 postchallenge, three mice per group were euthanized, and lungs and serum samples were collected. The entire left lung lobe was removed from each mouse and immersion-fixed in zinc formalin (Sigma-Aldrich) for 48 h. After the fixation period, samples were routinely processed and embedded in paraffin for subsequence histopathological evaluations. Right lung lobes were divided longitudinally in two, with one part placed in RNALater stabilization reagent (Sigma-Aldrich) and stored at −80 °C until RNA extraction, and the other part stored also at −80 °C until analysis of virus yields. Blood was collected by submandibular bleeding, maintained at 37 °C for 1 h, kept at 4 °C overnight, and centrifuged at 3600 rpm for 20 min at 4 °C to obtain the serum samples, which was then inactivated at 56 °C for 30 min and kept at −20 °C until use.

Moreover, K18-hACE2 mice previously vaccinated with one or two doses of MVA-CoV2-S (or control unvaccinated and unchallenged mice) (*n* = 5/group) were re-infected with SARS-CoV-2 at week 16 (7 weeks after the first SARS-CoV-2 infection) with a lethal dose (1 × 10^5^ PFU) of SARS-CoV-2 (MAD6 strain) by intranasal route in 50 μl of PBS. Mice were monitored for body weight change and mortality for 6 days post-rechallenge, moment at what control infected mice lost >25% of the initial body weight and have to be sacrificed. On day 6 post-rechallenge, all mice were euthanized, and lungs and serum samples were collected and processed similarly as above.

### Analysis of SARS-CoV-2 RNA by quantitative RT-PCR (RT-qPCR)

Lungs from K18-hACE2 mice were harvested at the indicated timepoints and stored in RNALater (Sigma-Aldrich) at −80 °C until homogenized with a gentleMACS dissociator (Miltenyi Biotec) in 2 ml of RLT buffer (Qiagen) plus β-mercaptoethanol (Sigma-Aldrich) and aliquoted. Then, 600 μl of homogenized lung tissue was used to isolate total RNA using the RNeasy Mini Kit (Qiagen), according to the manufacturer’s specifications. First-strand cDNA synthesis and subsequent real-time PCR were performed in one step using NZYSpeedy One-step RT-qPCR Master Mix (NZYTech), according to the manufacturer’s specifications using ROX as reference dye. SARS-CoV-2 viral RNA content was determined using previously validated set of primers and probes specific for the SARS-CoV-2 subgenomic RNA for the protein E, the genomic virus RNA dependent RNA polymerase (RdRp) gene and the cellular 28 S rRNA for normalization^47^ (Supplementary Table 1). Data were acquired with a 7500 real-time PCR system (Applied Biosystems) and analyzed with 7500 software v2.0.6. Relative RNA arbitrary units (A.U.) were quantified relative to the negative group (uninfected mice) and were performed using the 2-ΔΔCt method. All samples were tested in duplicate.

### Analysis of SARS-CoV-2 virus yields by plaque assay

Lungs from K18-hACE2 mice were harvested at the indicated timepoints, weighted, and stored directly at −80 °C until homogenized with a gentleMACS dissociator (Miltenyi Biotec) in 2 ml of PBS buffer and aliquoted. Then, undiluted and serial ten-fold dilutions of homogenized lung tissue were added in triplicate to Vero-E6 cell monolayers seeded in 12-well plates at 5 × 10^5^ cells/well and after 1 h of adsorption the inoculum was removed and plates were incubated at 37 °C, 5% CO_2_ in 2:1 DMEM 2X-4% fetal bovine serum (FBS):2% Agar. After 4 days, cells were fixed for 1 h with 10% formaldehyde (Sigma-Aldrich), and then the agarose was removed and plaques were visualized by adding 0.5% crystal violet (Sigma-Aldrich). SARS-CoV-2 titers were determined in PFUs per gram of lung tissue.

### Lung cytokine profile analysis by RT-qPCR

Reverse transcription of 1000 ng of RNA isolated as described above from lung homogenates of K18-hACE2 mice was performed with the QuantiTect reverse transcription kit (Qiagen, Hilden, Germany), according to the manufacturer’s recommendations. RT-qPCR was performed with a 7500 real-time PCR system (Applied Biosystems) using Power SYBR green PCR Master Mix (Applied Biosystems), as previously described^5,48^. The mRNA expression levels of the genes for IL-6, TNF-α, IL-10, IP-10 and IFN-γ were analyzed by real-time PCR with specific oligonucleotides (Supplementary Table 1). Specific gene expression was expressed relative to the expression of the cellular 28 S ribosomal RNA gene in fold change units using the 2 − ∆∆Ct method. All samples were tested in triplicate.

### Lung histopathology

The entire left lung lobe was removed from each K18-hACE2 mouse and immersion-fixed in zinc formalin (Sigma-Aldrich) for 48 h. After fixation period, samples were routinely processed and embedded in paraffin blocks that were then sectioned at 4 µm thickness on a microtome, mounted onto glass slides and routinely stained with hematoxylin and eosin (H&E). Lung sections were microscopically evaluated using an Olympus BX43 microscope by a single veterinary pathologist who was blinded to the identity and group of individual mice. To assess the character and severity of histopathological lesions, lung inflammation scoring parameters based on previous reports on SARS-CoV-2 infection in mouse models were used^23^. The histopathological parameters evaluated were the follows: capillary endothelial cell activation; alveolar hemorrhages; alveolar edema; perivascular edema; alveolar septal thickening (interstitial pneumonia); alveolar damage and hyaline membranes in alveoli; inflammatory cell infiltration in alveoli; bronchi/bronchioles with epithelial necrosis, detached epithelium or inflammatory cells in the lumen (bronchitis/bronchiolitis); peribronchial/peribronchiolar and perivascular mononuclear infiltrates; pneumocytes hyperplasia; cytopathic effect or syncytia; squamous metaplasia; uniform interstitial fibrosis; organized fibrotic tissue around the bronchi/bronchioles or intrabronchiolar (bronchiolitis obliterans) and pleural thickening. The histopathological parameters were graded following a semi-quantitative scoring system as follows: (0) no lesion; (1) minimal lesion; (2) mild lesion; (3) moderate lesion; (4) severe lesion. The cumulative scores of histopathological lesions provided the total score per animal. In each experimental group, the individual scores were used to calculate the group average. In addition, H&E-stained sections were visually scored 0–6 based on the percentage of lung area affected by inflammatory lesions as follows: 0% of the lung injured (score 0); <5% (score 1); 6–10% (score 2); 11–20% (score 3); 21–30% (score 4); 31–40% (score 5); >40% (score 6). In each experimental group, the individual scores were used to calculate the group average.

### Peptides and proteins

SARS-CoV-2 S peptide pools were used in the cellular immunogenicity analysis and were purchased from JPT Peptide Technologies (Berlin, Germany, catalog number PM-WCPV-S). The S peptide pools were divided into two groups spanning the S1 and S2 regions of the S protein, with each peptide pool containing 158 (S1) or 157 peptides (S2) as consecutive 15-mers overlapping by 11 amino acids.

The SARS-CoV-2 soluble S and RBD proteins were produced in mammalian cells as previously described^5^, and they were used to analyze by ELISA the levels of IgG antibodies in mice serum samples. The S sequence (residues 1–1208; Wuhan-Hu-1 strain, GenBank accession number MN908947.3) contained a T4 fibritin trimerization sequence, a Flag epitope and an 8xHis-tag at the C-terminus. In the S protein, the furin-recognition motif (RRAR) was replaced by the GSAS sequence, and it also contained the A942P, K986P, and V987P substitutions in the S2 portion. The S protein was purified by nickel-nitrilotriacetic acid (Ni-NTA) affinity chromatography from transfected cell supernatants and it was transferred to HEPES buffered saline (HBS) pH 7.5, during concentration or by size-exclusion chromatography (SEC).

The RBD proteins with the S residues 332–534 were produced with an N-terminal HA-tag (YPYDVPDYA) and fused to either a T4 fibritin trimerization sequence, a Flag epitope and an 8xHis-tag (RBD-TFH), or to the human IgG1 Fc region (RBD-Fc) at its C-terminus. RBD-Fc proteins with RBD substitutions in VoC were also produced: alpha (B.1.1.7; N501Y), beta (B.1.351; K417N-E484K-N501Y), gamma (P.1.; K417T-E484K-N501Y), delta (B.1.617.2; L452R-T478K), kappa (B.1.617.1; L452R-E484Q) and zeta (P.2; E484K). The RBD-TFH and RBD-Fc proteins were purified by affinity chromatography with Ni-NTA and IgSelect (GE Healthcare) columns, respectively.

### Immunogenicity study schedule in C57BL/6 mice

To evaluate the long-term memory immunogenicity of the MVA-CoV2-S vaccine candidate MVA-CoV2-S prime/MVA-CoV2-S boost immunization protocol was performed in female C57BL/6 mice (6–8 weeks old) as previously described^5^. Groups of animals (*n* = 5) received two doses of 1 × 10^7^ PFU of MVA-CoV2-S or MVA-WT (used as negative control mice) by intramuscular route in 100 μl of PBS (50 μl/leg) at 0 and 2 weeks. At 6 months after the last immunization, mice were sacrificed using CO_2_. Next, blood from each individual mouse was collected by cardiac puncture and processed as described above to obtain serum samples to analyze the titers of IgG antibodies, IgG isotypes, and neutralizing antibodies against SARS-CoV-2; the spleens of each group were pooled and processed to measure the long-term memory T-cell immune responses to the SARS-CoV-2 S antigen by ELISpot and ICS assays. No adverse effects were detected in immunized mice.

### ELISpot assay

The ELISpot assay was used to detect long-term memory SARS-CoV-2 S-specific IFNγ-secreting cells and was performed as previously described^5^. Briefly, 96-well nitrocellulose-bottom plates (Millipore) were covered with 75 μl/well of a solution of the rat anti-mouse IFN-γ monoclonal antibody (Pharmingen) at a concentration of 6 μg/ml in PBS. After incubating overnight at room temperature, the wells were washed three times with RPMI medium and blocked with RPMI-10% FCS for at least 1 h at 37 °C in a 5% CO_2_ atmosphere. After spleen processing, 10^6^ splenocytes per condition were restimulated in triplicate with a mix of 1 μg/ml of the SARS-CoV-2 S1 and S2 peptide pools (JPT Peptide Technologies) or with RPMI-10% FCS. The plates were incubated with the peptides for 48 h at 37 °C in a 5% CO_2_ atmosphere, washed five times with PBS-Tween 20, and incubated with 2 μg/ml of biotinylated rat anti-mouse IFN-γ monoclonal antibody XMG1.2 (Pharmingen) diluted in PBS-Tween 20 for 2 h at room temperature. The plates were then washed five times with PBS-Tween 20, and a 1:800 dilution of HRP-conjugated streptavidin (0.5 mg/ml; Sigma-Aldrich) was added. After 1 h at room temperature, it was washed three times with PBS-Tween 20 and two times with PBS, and finally, 1 μg/ml of the diaminobenzidine (DAB) substrate (Sigma-Aldrich) resuspended in 50 mM Tris-Cl (pH 7.5) and 0.015% H_2_O_2_ was added to develop the plates. The reaction was stopped by washing the plate with abundant water, and once it was dry, the spots were counted using the ELISpot Reader System ELR02 plate reader (AID Autoimmun Diagnostika GmbH) with the aid of AID ELISpot reader system software (Vitro).

### ICS assay

The magnitude, breadth, polyfunctionality, and memory phenotype of the long-term SARS-CoV-2 S-specific CD4^+^ and CD8^+^ T-cell memory immune responses expressing CD107a, and/or IFNγ, and/or TNFα, and/or IL-2 were analyzed by ICS as previously described^5^, in splenocytes stimulated with SARS-CoV-2 S1 and S2 peptide pools. After spleen processing, 4 × 10^6^ fresh splenocytes (depleted of red blood cells) were seeded on M96 plates and stimulated for 6 h in complete RPMI 1640 medium supplemented with 10% FCS containing 1 μl/ml Golgiplug (BD Biosciences) to inhibit cytokine secretion, 1X monensin (eBioscience, Thermo Fisher Scientific), anti-CD107a–fluorescein isothiocyanate (FITC) (BD Biosciences), and the SARS-CoV-2 S1 and S2 peptide pools (JPT Peptide Technologies, 1 μg/ml). Cells were then washed, stained for surface markers, fixed, permeabilized (Cytofix/Cytoperm kit; BD Biosciences), and stained intracellularly with the appropriate fluorochromes. Dead cells were excluded using the violet LIVE/DEAD stain kit (Invitrogen). The fluorochrome-conjugated antibodies used for functional analyses were CD3-phycoerythrin (PE)-CF594, CD4-allophycocyanin (APC)-Cy7, CD8-V500, IFN-γ–PE-Cy7, TNF-α–PE, and IL-2–APC. In addition, the antibodies used for memory phenotypic analyses were CD62L-Alexa Fluor 700 and CD127-peridinin chlorophyll protein (PerCP)-Cy5.5. All antibodies were from BD Biosciences. Cells were acquired with a Gallios flow cytometer (Beckman Coulter), and analyses of the data were performed with the FlowJo software version 10.4.2 (Tree Star), as previously described^5^. Gating strategy is shown in Supplementary Fig. 4.

### ELISA

The titers of binding IgG, IgG1, and IgG2c anti-S and -RBD antibodies in individual or pooled sera samples from immunized K18-hACE2 or C57BL/6 mice were measured by ELISA, as previously described^5^. Briefly, 96-well Nunc MaxiSorp plates were coated with 50 μl of purified recombinant SARS-CoV-2 S or RBD proteins at a concentration of 2 μg/ml in PBS at 4 °C overnight. Plates were washed with PBS (Gibco-Life Technologies) supplemented with 0.05% Tween 20 (PBS-Tween) and blocked with 5% milk in PBS for 2 h at room temperature (RT). Individual or pooled serum samples from each immunization group were diluted in PBS-Tween-1% milk, added to plates, and incubated for 1.5 h at RT. Plates were then washed, and secondary HRP-conjugated goat anti-mouse IgG, IgG1, or IgG2c antibodies (Southern Biotech; all diluted 1:1000 in PBS-Tween-1% milk) were added and incubated for 1 h at RT. Plates were washed, the TMB substrate (Sigma-Aldrich) was added, and the reaction was stopped by adding 1 M H_2_SO_4_. Absorbance was read at 450 nm. Total binding IgG titers were measured as the last serum dilution that gives an absorbance value at 450 nm at least three times higher than the absorbance of a naive serum. Purified RBD-Fc proteins without (WT, Wuhan) or with mutations in VoC (alpha, beta, gamma, delta, kappa and zeta) were used in ELISA assays to determine the RBD-specific IgG antibody titers. Similar amounts of plastic-bound RBD-Fc proteins were used, as determined with an anti-HA antibody (2.5–0.0025 μg/ml) that recognized an HA tag at the RBD N-terminus. Differences in the HA antibody signal at 450 nm were used to standardize the anti-mouse IgG binding to RBD variants with respect to the WT before IgG titer determination.

### SARS-CoV-2 neutralization

Capacity of the sera obtained from K18-hACE2 or C57BL/6 mice immunized with MVA-CoV2-S to neutralize SARS-CoV-2 virus was determined using retrovirus-based pseudoparticles expressing SARS-CoV-2 S protein, as previously described^5^. Pseudotyped viruses were produced by transfection of a DNA plasmid expressing the full-length S protein^5^, together with packaging plasmids expressing the structural proteins Gag and Pol (kindly provided by F. L. Cosset [INSERM, Lyon, France]) in HEK-293T cells, as previously described^49^. Briefly, Vero-E6 cells were seeded in 96-well plates at 10,000 cells/well in DMEM-10% FBS medium. Twenty-four hours post seeding, a mix of retrovirus-based pseudoparticles expressing SARS-CoV-2 S protein and serial two-fold dilutions of mouse serum samples were preincubated for 1 h at 37 °C and then added to the cells in triplicates in DMEM-2% FBS medium. The medium was replaced at 24 h postinfection, and 24 h later cells were lysed in passive lysis buffer (Promega) and luciferase activity was measured in a luminometer (Thermo Appliskan multimode microplate reader; Thermo Fisher Scientific). Titers of neutralizing antibodies were determined as the highest serum dilution which resulted in a 50% reduction of luciferase units (neutralizing titer 50 [NT_50_]) compared with pseudotyped viruses not incubated with serum.

Moreover, live-virus SARS-CoV-2 neutralizing antibodies were also measured using a microneutralization test (MNT) assay in a BSL-3 laboratory at the CNB-CSIC. Serially two-fold diluted mouse serum samples in DMEM-2% FBS medium were incubated at a 1:1 ratio with 100 TCID50 of SARS-CoV-2 MAD6 isolate in 96-well tissue culture plates for 1 h at 37 °C. Then, mixtures of serum samples and SARS-CoV-2 virus were added in duplicate to Vero-E6 cell monolayers seeded in 96-well plates at 30,000 cells/well, and plates were incubated at 37 °C, in a 5% CO_2_ incubator for 3 days. Then, cells were fixed with 10% formaldehyde for 1 h and stained with crystal violet. When plates were dried, crystal violet was diluted in H_2_O-10% SDS and optical density was measured in a luminometer at 570 nm. NT_50_ titers were calculated as the reciprocal dilution resulting in 50% inhibition of cell death following a methodology previously described^50^. A WHO International Standard containing pooled plasma obtained from eleven individuals recovered from SARS-CoV-2 infection (NIBSC code: 20/136) was used for the calibration and harmonization of both serological assays detecting anti-SARS-CoV-2 neutralizing antibodies.

### Neutralization of SARS-CoV-2 variants of concern

Capacity of serum samples obtained from K18-hACE2 or C57BL/6 mice immunized with MVA-CoV2-S to neutralize different SARS-CoV-2 VoC was tested by using SARS-CoV-2 pseudotyped Vesicular Stomatitis Viruses (VSV) expressing S protein. SARS-CoV-2 S protein pseudotyped rVSV-luc recombinant viruses (PSV) were produced as described elsewhere^51^. SARS-CoV-2 S variants used were S_614D, S_614G, alpha (B.1.1.7), beta (B.1.351), gamma (P.1), and delta (B.1.617.2). SARS-CoV-2 S mutant D614G was generated by site-directed mutagenesis (Q5 Site-Directed Mutagenesis Kit; New England Biolabs) following the manufacturer’s instructions and using as an input DNA a pcDNA3.1 expression vector encoding SARS-CoV-2 S_614D^5^. SARS-CoV-2 VoC alpha (B.1.1.7; GISAID: EPI_ISL_608430), beta (B.1.351; GISAID: EPI_ISL_712096), gamma (P.1; GISAID: EPI_ISL_833140) and delta (B.1.617.2; GISAID: EPI_ISL_1970335) were optimized, synthesized and cloned into pcDNA3.1 by GeneArt (Thermo Fisher Scientific, GeneArt GmbH, Regensburg, Germany).

The neutralization activity of serum samples was tested by triplicates at dilutions 1:200 to 1:12800. For neutralization experiments, viruses-containing transfection supernatants were normalized for infectivity to an MOI of 0.5–1 and incubated with the dilutions of serum samples at 37 °C for 1 h in 96-well plates. After the incubation time, 2 × 10^4^ Vero-E6 cells were seeded onto the virus-serum mixture and incubated at 37 °C for 24 h. Cells were then lysed, assayed for luciferase expression and NT_50_ titers were calculated.

### Correlation analysis

Correlates of protection analysis were performed using GraphPad Prism 9.2.0 (GraphPad Software) following the methodology previously described^52^. Mice were classified either as protected or non-protected from SARS-CoV-2 infection, defined as a lung viral load of either below or above 5 × 10^2^ PFU/g, respectively. Simple logistic regression models were built from binding anti-S IgG and neutralizing antibody data grouped from different immunization regimens, with protection outcome as the dependent variable, and Log10 transformed endpoint antibody and NT_50_ titers before challenge as the independent variable. Correlations between Log10 transformed endpoint antibody and NT_50_ titers before challenge and data related to protection (log10 transformed viral load, gRNA, and lung inflammation scores) were assessed by two-sided Pearson correlation coefficient. *R*^2^ values > −0.5 and −0.75 indicate a moderate and strong correlation, respectively. *P*-values < 0.05 were considered significant.

### Statistical procedures

For statistical analysis one-way ANOVA of transform data followed by post-hoc Student’s *t*-test comparisons was used to establish the differences between the two groups. Statistical analysis of the ICS assay results was realized as previously described^53^, using an approach that corrects measurements for background response, calculating confidence intervals and *p*-values. The statistical significances are indicated as follows: *p < 0.05; **p < 0.005; ***p < 0.001.

### Reporting summary

Further information on research design is available in the Nature Research Reporting Summary linked to this article.

## Supplementary information

REPORTING SUMMARY

Supplementary information

## Data Availability

The datasets generated and/or analyzed during the current study are available from the corresponding author on reasonable request.
