# Supplementary material for: Full efficacy and long-term immunogenicity induced by the SARS-CoV-2 vaccine candidate MVA-CoV2-S in mice
Source: NPJ Vaccines. 2022 Feb 9;7:17. doi: 10.1038/s41541-022-00440-w (PMC8828760; doi:10.1038/s41541-022-00440-w)
Supplement: Supplementary file 1 — REPORTING SUMMARY [file 41541_2022_440_MOESM1_ESM.pdf]

nature portfolio

Corresponding author(s): DBPR. NPJVACCINES-01557R

Last updated by author(s): Nov 24, 2021

Reporting Summary

Nature Portfolio wishes to improve the reproducibility of the work that we publish. This form provides structure for consistency and transparency in reporting. For further information on Nature Portfolio policies, see our Editorial Policies and the Editorial Policy Checklist.

Statistics

For all statistical analyses, confirm that the following items are present in the figure legend, table legend, main text, or Methods section.

n/a	Confirmed	
☐	☒	The exact sample size (*n*) for each experimental group/condition, given as a discrete number and unit of measurement
☐	☒	A statement on whether measurements were taken from distinct samples or whether the same sample was measured repeatedly
☐	☒	The statistical test(s) used AND whether they are one- or two-sided<br>*Only common tests should be described solely by name; describe more complex techniques in the Methods section.*
☒	☐	A description of all covariates tested
☒	☐	A description of any assumptions or corrections, such as tests of normality and adjustment for multiple comparisons
☐	☒	A full description of the statistical parameters including central tendency (e.g. means) or other basic estimates (e.g. regression coefficient) AND variation (e.g. standard deviation) or associated estimates of uncertainty (e.g. confidence intervals)
☐	☒	For null hypothesis testing, the test statistic (e.g. *F*, *t*, *r*) with confidence intervals, effect sizes, degrees of freedom and *P* value noted<br>*Give P values as exact values whenever suitable.*
☒	☐	For Bayesian analysis, information on the choice of priors and Markov chain Monte Carlo settings
☒	☐	For hierarchical and complex designs, identification of the appropriate level for tests and full reporting of outcomes
☐	☒	Estimates of effect sizes (e.g. Cohen's *d*, Pearson's *r*), indicating how they were calculated

Our web collection on statistics for biologists contains articles on many of the points above.

Software and code

Policy information about availability of computer code

Data collection	No software was used
Data analysis	GraphPad Prism. Versions 8.0.1 and 9.2.0

For manuscripts utilizing custom algorithms or software that are central to the research but not yet described in published literature, software must be made available to editors and reviewers. We strongly encourage code deposition in a community repository (e.g. GitHub). See the Nature Portfolio guidelines for submitting code & software for further information.

Data

Policy information about availability of data

All manuscripts must include a data availability statement. This statement should provide the following information, where applicable:

- Accession codes, unique identifiers, or web links for publicly available datasets
- A description of any restrictions on data availability
- For clinical datasets or third party data, please ensure that the statement adheres to our policy

The datasets generated and/or analyzed during the current study are available from the corresponding author on reasonable request.

Field-specific reporting

Please select the one below that is the best fit for your research. If you are not sure, read the appropriate sections before making your selection.

☒ Life sciences ☐ Behavioural & social sciences ☐ Ecological, evolutionary & environmental sciences

For a reference copy of the document with all sections, see nature.com/documents/nr-reporting-summary-flat.pdf

Life sciences study design

All studies must disclose on these points even when the disclosure is negative.

Sample size	The statistical test carried out is the T-student test. We have applied the G power software to calculate the sample size for a T-test of two independent groups, using the following values: α = 0.05; β = 0.05; Sx = Sy (group standard deviation) = 0.33 and µd (effect size) = 0.8. With these values we obtained a final value of n = 5 mice / group.<br>Thus, groups of mice of at least 5 mice were used.
Data exclusions	No data were excluded.
Replication	All analysis were performed at least by duplicate.
Randomization	Mice were randomized to included them in the different experimental groups.
Blinding	All samples were blinding analyzed.

Reporting for specific materials, systems and methods

We require information from authors about some types of materials, experimental systems and methods used in many studies. Here, indicate whether each material, system or method listed is relevant to your study. If you are not sure if a list item applies to your research, read the appropriate section before selecting a response.

Materials & experimental systems

n/a	Involved in the study	
☐	☒	Antibodies
☐	☒	Eukaryotic cell lines
☒	☐	Palaeontology and archaeology
☐	☒	Animals and other organisms
☒	☐	Human research participants
☒	☐	Clinical data
☒	☐	Dual use research of concern

Methods

n/a	Involved in the study	
☒	☐	ChIP-seq
☐	☒	Flow cytometry
☒	☐	MRI-based neuroimaging

Antibodies

Antibodies used	Included in the manuscript. Methods section.
Validation	Antibodies were validated by the manufacturers and in our Flow Cytometry department at the CNB-CSIC.

Eukaryotic cell lines

Policy information about cell lines

Cell line source(s)	Included in the manuscript. Methods section. Cells derived from ATCC.<br>Vero-E6 cells (ATCC catalog no. CRL-1586).
Authentication	Authentication was performed, as cells were derived directly from ATCC.
Mycoplasma contamination	All cell lines were tested negative for mycoplasma.
Commonly misidentified lines<br>(See ICLAC register)	*Name any commonly misidentified cell lines used in the study and provide a rationale for their use.*

Animals and other organisms

Policy information about studies involving animals; ARRIVE guidelines recommended for reporting animal research

Laboratory animals	Included in the manuscript. Methods section.<br>- Female transgenic K18-hACE2 mice. 034860-B6.Cg-Tg(K18-ACE2)2Prlmn/J<br>- Female C57BL/6OlaHsd mice.
Wild animals	No wild animals were used.
Field-collected samples	No field-collected sample were used.
Ethics oversight	Included in the manuscript. Methods section.<br>PROEX 49/20, 169.4/20 and 161.5/20. Comunidad de Madrid, Spain

Note that full information on the approval of the study protocol must also be provided in the manuscript.

Flow Cytometry

Plots

Confirm that:

☒ The axis labels state the marker and fluorochrome used (e.g. CD4-FITC).

☒ The axis scales are clearly visible. Include numbers along axes only for bottom left plot of group (a 'group' is an analysis of identical markers).

☒ All plots are contour plots with outliers or pseudocolor plots.

☒ A numerical value for number of cells or percentage (with statistics) is provided.

Methodology

Sample preparation	Included in the manuscript. Methods section.
Instrument	Gallios flow cytometer (Beckman Coulter)
Software	FlowJo software version 10.4.2 (Tree Star)
Cell population abundance	Not applicable
Gating strategy	*Describe the gating strategy used for all relevant experiments, specifying the preliminary FSC/SSC gates of the starting cell population, indicating where boundaries between "positive" and "negative" staining cell populations are defined.*

☐ Tick this box to confirm that a figure exemplifying the gating strategy is provided in the Supplementary Information.
