# Supplementary material for: Full efficacy and long-term immunogenicity induced by the SARS-CoV-2 vaccine candidate MVA-CoV2-S in mice
Source: NPJ Vaccines. 2022 Feb 9;7:17. doi: 10.1038/s41541-022-00440-w (PMC8828760; doi:10.1038/s41541-022-00440-w)
Supplement: Supplementary file 2 — Supplementary information [file 41541_2022_440_MOESM2_ESM.pdf]

Supplementary information

Full efficacy and long-term immunogenicity induced by the SARS-CoV-2 vaccine candidate MVA-CoV2-S in mice

Adrián Lázaro-Frías[1,2,7], Patricia Pérez[1,2,7], Carmen Zamora[1], Pedro J. Sánchez-Cordón[3], María Guzmán[4], Joanna Luczkowiak[5], Rafael Delgado[5,6], José M. Casasnovas[4], Mariano Esteban[1,] 🖂 & Juan García-Arriaza[1,2,] 🖂

[1] Department of Molecular and Cellular Biology, Centro Nacional de Biotecnología (CNB), Consejo Superior de Investigaciones Científicas (CSIC), 28049 Madrid, Spain. [2] Centro de Investigación Biomédica en Red de Enfermedades Infecciosas (CIBERINFEC), Madrid, Spain. [3] Pathology Department, Centro de Investigación en Sanidad Animal (CISA), Instituto Nacional de Investigación y Tecnología Agraria y Alimentaria (INIA), Consejo Superior de Investigaciones Científicas (CSIC), 28130 Valdeolmos, Madrid, Spain. [4] Department of Macromolecular Structures, Centro Nacional de Biotecnología (CNB), Consejo Superior de Investigaciones Científicas (CSIC), 28049 Madrid, Spain. [5] Instituto de Investigación Hospital Universitario 12 de Octubre (imas12), 28041 Madrid, Spain. [6] Universidad Complutense School of Medicine, 28040 Madrid, Spain. [7] These authors contributed equally: Adrián Lázaro-Frías, Patricia Pérez. 🖂email: mesteban@cnb.csic.es; jfgarcia@cnb.csic.es

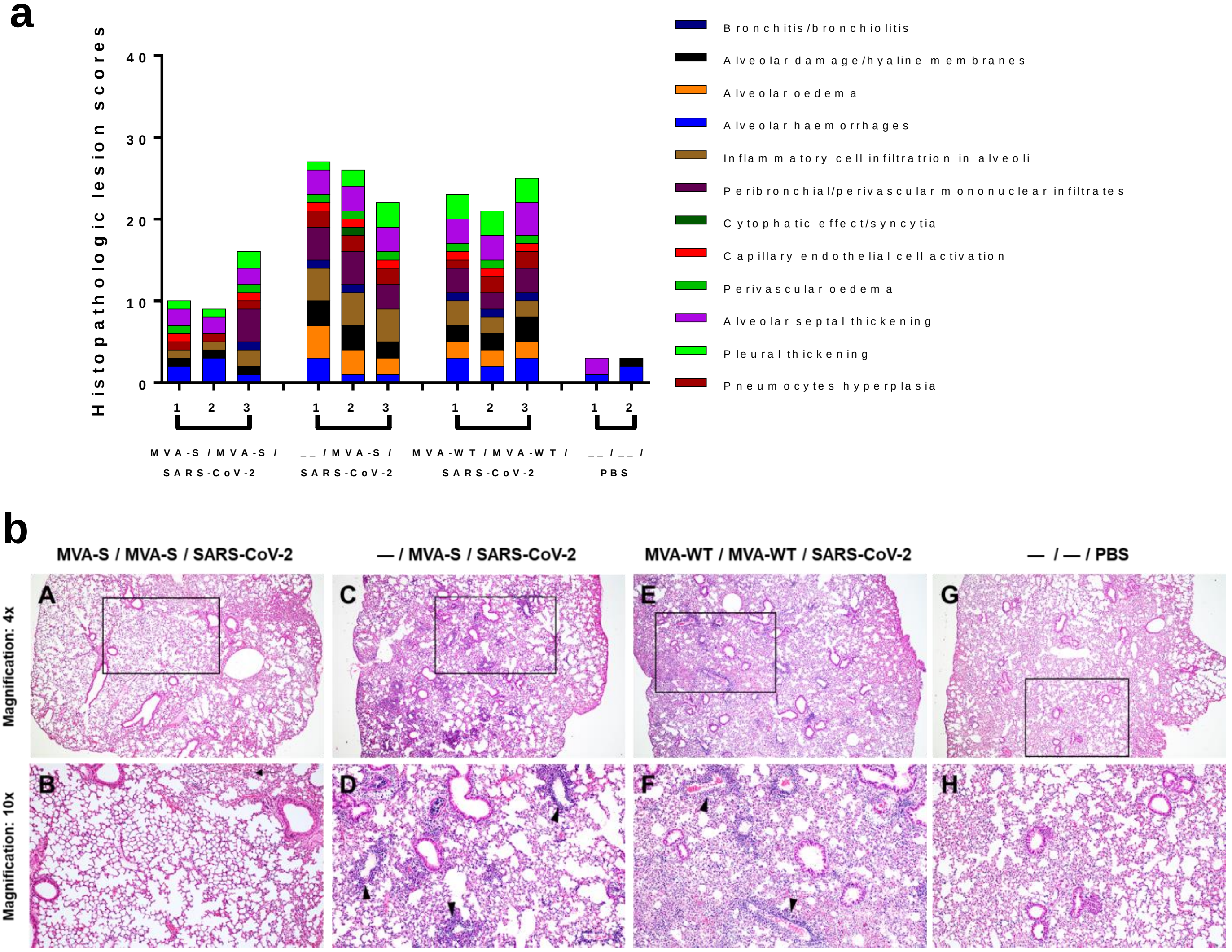

Supplementary Figure 1. Lung histopathological analysis of mice vaccinated with MVA-S and challenged with SARS-CoV-2. (a) Individual representation of the cumulative histopathological scores examined in lung samples (n = 3/group) obtained at 4 days postchallenge from mice vaccinated and infected as indicated in Fig. 1a. Lung histopathologic lesions observed are displayed in the graph by different colours. Score of histopathologic lesions (y-axis); mice evaluated in each experimental group (x-axis). **(b)** Representative lung histopathological sections (H&E staining) from K18-hACE2 mice euthanized at day 4 postchallenge. A general view of the lung area (magnification: 4x) along with histopathological details from selected lung areas (black boxes) have been displayed (magnification: 10x). Before challenging, K18-hACE2 mice were previously vaccinated with two doses (A, B) and one dose of MVA-S (C, D), respectively. K18-hACE2 mice immunized with two doses of MVA-WT before the challenge (E, F) and unvaccinated mock-infected K18-hACE2 mice (G, H) were also included as control infected and control non-infected group, respectively. Mice vaccinated with two doses of MVA-S (A, B) displayed less extend and severe lung inflammatory lesions compared to mice immunized with one dose of MVA-S (C, D) or control MVA-WT inoculated mice (E, F). Mice vaccinated with two doses of MVA-S (B) only displayed focal thickening of the alveolar septae (arrow), and occasional presence of inflammatory cells within the alveoli. However, mice immunized with one dose of MVA-S (D) or control MVA-WT inoculated mice (F) showed more severe diffuse thickening of the alveolar septae, higher presence of mononuclear cell infiltrates within alveolar spaces as well as the presence of larger multifocal perivascular and peribronchiolar mononuclear infiltrates (arrowheads). Unvaccinated mock-infected mice (H) did not display remarkable inflammatory lesions.

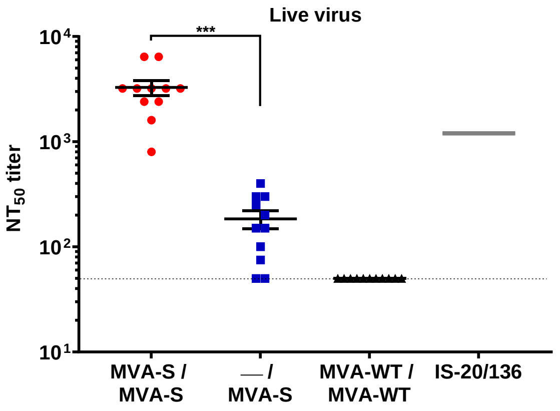

Supplementary Figure 2. SARS-CoV-2 neutralizing antibody titers. Titers were evaluated in individual mouse serum samples collected from immunized K18-hACE2 mice at 10 days postboost, using a live-virus microneutralization assay. The titers (NT_{50}) were calculated as the reciprocal dilution resulting in 50% inhibition of cell death. Mean NT_{50} values and SEM for each immunization group is represented. Dotted line shows the detection limit. Student's t-test: ***, $P < 0.001$. The NIBSC 20/136 international standard containing pooled plasma obtained from 11 individuals recovered from SARS-CoV-2 infection is included.

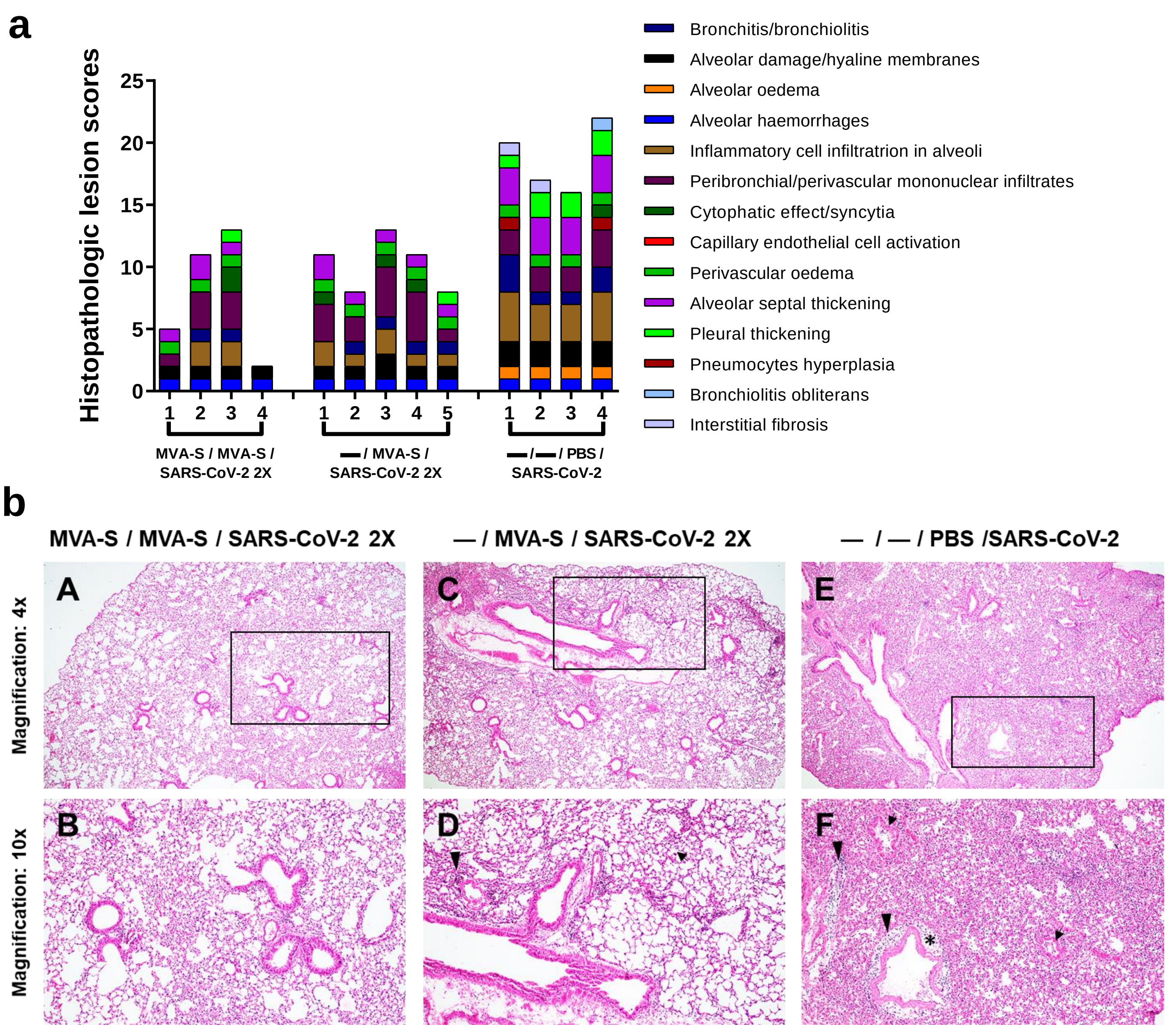

Supplementary Figure 3. Lung histopathological analysis of mice vaccinated with MVA-S and re-challenged with SARS-CoV-2. (a) Individual representation of the cumulative histopathological scores examined in lung samples obtained at day 6 days post-rechallenge from mice vaccinated and infected as indicated in Fig. 4a. Lung histopathologic lesions observed are displayed in the graph by different colours. Score of histopathologic lesions (y-axis); Mice evaluated in each experimental group (x-axis). **(b)** Representative lung histopathological sections (H&E staining) from surviving K18-hACE2 mice immunized with one or two doses of MVA-S. Surviving mice were challenged with a second dose of SARS-CoV-2 at week 16 and euthanized at day 6 post challenge. Mock vaccinated K18-hACE2 mice were also challenged and included as control infected group. A general view of the lung area (magnification: 4x) along with histopathological details from selected lung areas (black boxes) have been displayed (magnification: 10x). Mice vaccinated with two doses of MVA-S (A, B) as well as mice vaccinated with one dose (C, D) displayed significantly lower lung lesion scores and lower percentages of lung area with lesions compared to control infected mice (E, F). Some mice vaccinated with two doses of MVA-S did not show remarkable histopathological lesions (B). Mice vaccinated with one dose of MVA-S (D), displayed only some mild lesions such as occasional presence of inflammatory cells, mainly macrophages, in alveoli (arrows), mild focal alveolar septal thickening and perivascular and peribronchiolar cuffs populated by mononuclear cells (arrowheads). On the contrary, mice belonging to the control infected group (F) displayed severe lung inflammatory changes characterized by the presence of marked perivascular edema (star), perivascular and peribronchiolar infiltrates (arrowheads), severe diffuse inflammatory infiltrates in alveolar spaces accompanied by generalized alveolar septae thickening and inflammatory cells along with detached epithelium within the lumen of bronchioles (arrows).

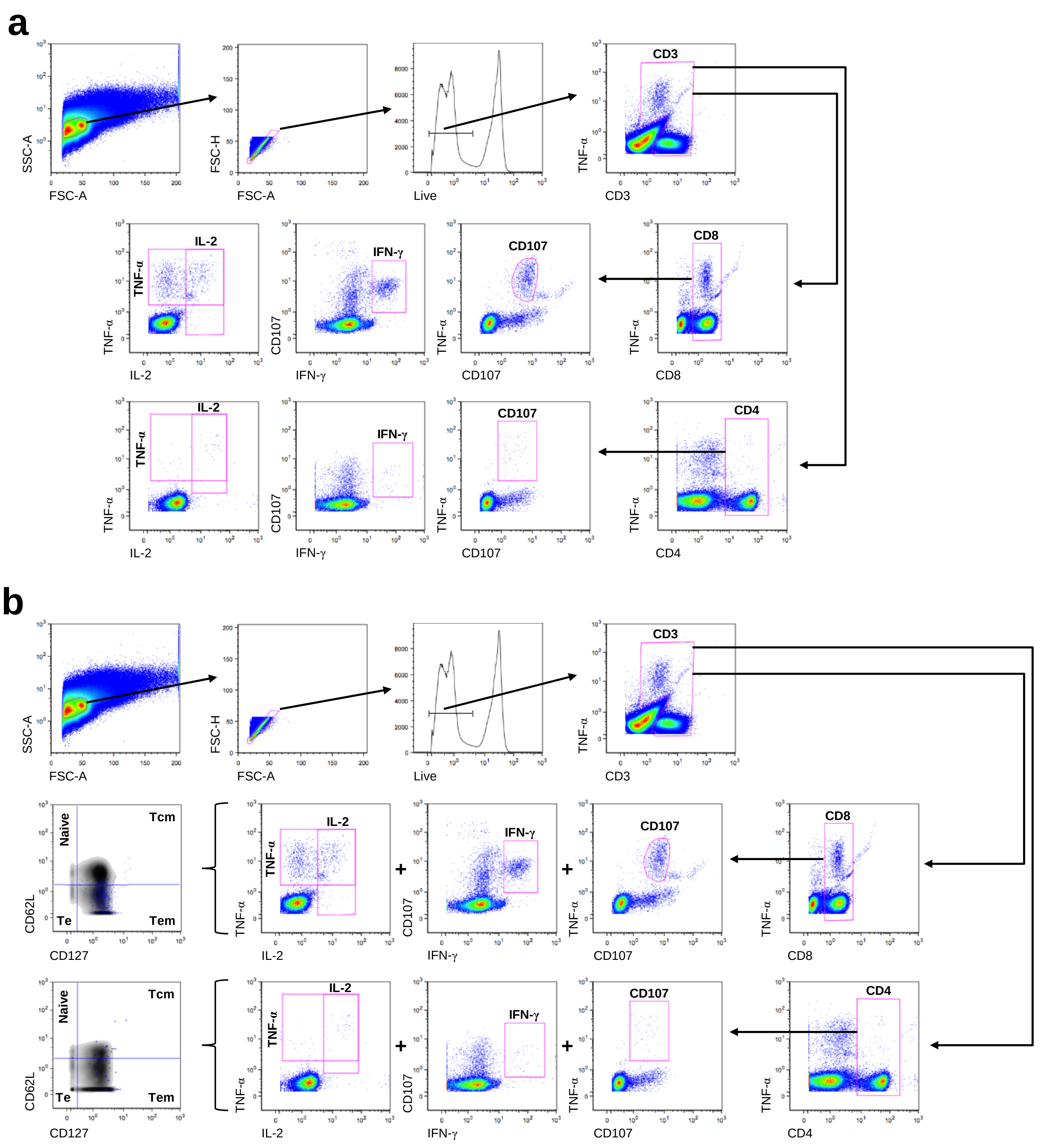

Supplementary Figure 4. Gating strategy used to analyze SARS-CoV-2 S-specific T-cell memory immune responses induced in mice vaccinated with MVA-S. (a) Gating strategy performed to detect S-specific CD4^{+} and CD8^{+} T cells expressing CD107a and/or producing IFN-γ and/or TNF-α and/or IL-2 in mice immunized with MVA-S. Representative plots of responses against S1 peptide pool are shown. Data obtained were analyzed by FlowJo, processed by subtracting background from unstimulated cells and then represented in Fig. 6b-c,e. **(b)** Gating strategy performed to detect different T memory phenotypes of S-specific CD4^{+} and CD8^{+} T cells expressing CD107a and/or producing IFN-γ and/or TNF-α and/or IL-2 in mice immunized with MVA-S. Representative plots of responses against S1 peptide pool are shown. Data obtained were analyzed by FlowJo, processed by subtracting background from unstimulated cells and then represented in Fig. 6d.

Supplementary Table 1. Primers used for the analysis by RT-qPCR of proinflammatory cytokines in lung homogenates.

Oligonucleotide name	Sequence (5' → 3')
IL-6 sense	CCTCTGGTCTTCTGGAGTACC
IL-6 antisense	ACTCCTTCTGTGACTCCAGC
TNF-α sense	ATGAGCACAGAAAGCATGA
TNF-α antisense	AGTGACAGAAGAGGGTGGT
IL-10 sense	AGCCTTATCGGAAATGATCCAGT
IL-10 antisense	GGCCTTGTAGACACCTTGGT
IP-10 sense	CCAAGTGCTGCCGTCATTTTC
IP-10 antisense	GGCTCGCAGGGATGATTTCAA
IFN-γ sense	CTGCCACGGCACAGTCATTG
IFN-γ antisense	TGCATCCTTTTTCGCCTTGC
E_sarbeco F1	ACAGGTACGTTAATAGTTAATAGCGT
E_Sarbeco R2	ATATTGCAGCAGTACGCACACA
E_Sarbeco P1	[6FAM] ACACTAGCCATCCTTACTGCGCTTCG [TAM]
RdRp-SARSr-F2	GTGARATGGTCATGTGTGGCGG
RdRp-SARSr-R1	CARATGTTAAASACACTATTAGCATA
RdRp-SARS-P2	[6FAM] CAGGTGGAACCTCATCAGGAGATGC [TAM]
Chlorocebus 28S forward	GGCGAAAGACTAATCGAACCAT
Chlorocebus 28S reverse	CGAGAGCGCCAGCTATCCT
Chlorocebus 28S Probe	[JOE] TAGTAGCTGGTTCCCTCCGAAGTTTCCCT [TAM]
